# The m^6^A methyltransferase METTL14 promotes cell proliferation via SETBP1-mediated activation of PI3K-AKT signaling pathway in myelodysplastic neoplasms

**DOI:** 10.1038/s41375-024-02350-3

**Published:** 2024-07-25

**Authors:** Lingxu Jiang, Yudi Zhang, Jiejing Qian, Xinping Zhou, Liya Ma, Shuanghong Zhu, Lu Wang, Wei Wang, Wenli Yang, Yingwan Luo, Wei Lang, Gaixiang Xu, Yanling Ren, Chen Mei, Li Ye, Qi Zhang, Xiaozhen Liu, Jie Jin, Jie Sun, Hongyan Tong

**Affiliations:** 1 Department of Hematology, The First Affiliated Hospital, Zhejiang University School of Medicine, Hangzhou, Zhejiang China; 2 Myelodysplastic Syndromes Diagnosis and Therapy Center, The First Affiliated Hospital, Zhejiang University School of Medicine, Hangzhou, Zhejiang China; 3 Zhejiang Provincial Key Lab of Hematopoietic Malignancy, Zhejiang University, Hangzhou, Zhejiang China; 4 Cancer Center, Zhejiang University, Hangzhou, Zhejiang China

**Keywords:** Oncogenes, Myelodysplastic syndrome, Oncogenesis

## Abstract

N6-methyladenosine (m^6^A) is the most prevalent epitranscriptomic modification in mammalian mRNA. Recent studies have revealed m^6^A is involved in the pathogenesis of various malignant tumors including hematologic neoplasms. Nevertheless, the specific roles of m^6^A modification and m^6^A regulators in myelodysplastic neoplasms (MDS) remain poorly understood. Herein, we demonstrated that m^6^A level and the expression of m^6^A methyltransferase METTL14 were elevated in MDS patients with bone marrow blasts ≥5%. Additionally, m^6^A level and METTL14 expression were upregulated as the disease risk increased and significantly associated with adverse clinical outcomes. Knockdown of METTL14 inhibited cell proliferation and colony formation ability of MDS cells. Moreover, in vivo experiments showed METTL14 knockdown remarkably reduced tumor burden and prolonged the survival of mice. Mechanistically, METTL14 facilitated the m^6^A modification of SETBP1 mRNA by formation of METTL3-METTL14 complex, leading to increased stabilization of SETBP1 mRNA and subsequent activation of the PI3K-AKT signaling pathway. Overall, this study elucidated the involvement of the METTL14/m^6^A/SETBP1/PI3K-AKT signaling axis in MDS, highlighting the therapeutic potential of targeting METTL3-METTL14 complex-mediated m^6^A modification for MDS therapy.

## Introduction

The myelodysplastic neoplasms (MDS), formerly known as myelodysplastic syndromes, is a group of malignant clonal neoplasms that exhibit chronic cytopenia, morphologic dysplasia of bone marrow, and risks for progressing to acute myeloid leukemia (AML) [1]. Epigenetic abnormalities play a crucial role in the progression of MDS, and epigenetic silencing of tumor suppressor genes caused by DNA methylation is thought to be a driving event in the pathogenesis of MDS [2, 3]. Consequently, the hypomethylating agents (HMAs) have been used in the treatment of higher-risk MDS patients for nearly a decade [4–6]. However, the efficacy of HMAs falls short of satisfying the clinical treatment requirements [7–11]. Therefore, it is imperative to explore novel molecular mechanisms and develop efficacious agents for MDS patients.

N6-methyladenosine (m^6^A), which is the most prevalent epitranscriptomic modification in mammalian mRNA, refers to the methylation of the N6 position on adenosine [12, 13]. Unlike DNA methylation, which regulates gene expression at the transcriptional level, m^6^A is a post-transcriptional modification that modulates gene expression by controlling mRNA stability, translation, splicing, and degradation [12, 13]. Accumulating evidence suggested that dysregulated m^6^A modification and m^6^A regulators played crucial roles in the development and progression of various cancers [14–17] including malignant hematological diseases [15–19]. The m^6^A modification process is predominantly catalyzed by the METTL3-METTL14 heterodimeric methyltransferase complex. METTL3 contains the active methyltransferase domain responsible for converting adenosine (A) to m^6^A, whereas METTL14 plays a crucial role in facilitating the catalytic activity of METTL3 by recognition and binding of target RNA substrates [12, 13]. Both METTL3 and METTL14 had been linked to the initiation and maintenance of AML [15, 16]. Specifically, METTL3 was found to be involved in the initiation and progression of AML in an m^6^A-dependent manner [15]. METTL14 was highly expressed in specific subtypes of AML patients, and facilitated AML tumorigenesis through the regulation of crucial targets such as MYB and MYC [16]. However, the biological significance of m^6^A modification and the relevant regulatory mechanisms in MDS remain elusive.

SETBP1, a nuclear protein, physically interacts with the oncoprotein SET and safeguards it against protease cleavage [20]. Growing evidence indicated that SETBP1 played an important role in the development and progression of various malignant tumors [20–22]. Overexpression of SETBP1 had been identified in 30% of patients with AML and chronic myeloid leukemia (CML) patients in blast crisis [23]. Activation of SETBP1 had been shown to contribute to the maintenance of leukemia stem cell self-renewal and the promotion of leukemia development through the inhibition of PP2A in myeloid leukemias [21]. However, the specific roles and underlying mechanisms of SETBP1 in MDS have yet to be explored.

In this study, we observed m^6^A modification and METTL14 expression were elevated in MDS patients with bone marrow blasts ≥5%, and positively associated with higher IPSS-R risks and adverse clinical outcomes. Moreover, we demonstrated that METTL14 promoted MDS cell proliferation by enhancing the expression of the downstream target SETBP1 through formation of METTL3-METTL14 heterodimer, which subsequently activating the PI3K-AKT signaling pathway.

## Methods

### Human samples from our center

Bone marrow mononuclear cells (BM-MNCs) were collected from newly diagnosed MDS patients as well as healthy donors, and isolated using Ficoll. CD34^+^ cells from BM-MNCs were purified by CD34 beads. A total of 253 MDS patients from our center were included in this study. Among them, BM-MNCs from 29 MDS patients and 11 healthy donors were used for the assessment of the global m^6^A level; BM-MNCs from 221 MDS patients and 29 healthy donors were collected for q-PCR analysis of METTL14 expression; isolated CD34^+^ cells from BM-MNCs of 16 MDS patients and 10 healthy donors were processed for RNA-seq analysis; BM-MNCs of 152 MDS patients and 8 healthy donors were processed for q-PCR analysis of SETBP1 expression. Patients diagnosed as de novo MDS at our center were satisfied the diagnostic criteria of the 2022 World Health Organization (WHO) classification [24]. Patients with a history of other malignancies, exposure to radiation, or previous anti-tumor treatments were excluded from the study. Bone marrow samples were obtained at the time of initial diagnosis, prior to treatment. The detailed characteristics including gender, age, 2022 WHO classification, the percentages of bone marrow blasts, the revised International Prognostic Scoring System (IPSS-R) scores, the karyotypes, and the follow-up treatment of each MDS patient were showed in the Supplementary Table 1. The baseline characteristics of the patients’ cohorts were summarized in the Supplementary Table 2.

### Publicly available MDS cohort

A publicly available MDS cohort (GSE58831) containing gene expression profile of bone marrow CD34^+^ cells from MDS patients and healthy donors was used in our study. The clinical characteristics, gene mutation status, and gene expression data were downloaded from Gene Expression Omnibus (GEO) databases at www.ncbi.nlm.nih.gov/geo/. We excluded the patients who lacked the data of gene expression, gene mutation status, and survival. Also, patients with a diagnosis of CMML or RARS-T were excluded. Finally, a total of 113 MDS patients were involved in our study. The baseline characteristics of the patients included were summarized in the Supplementary Table 3.

### In vivo experiments

MDS-L-luciferase (MDS-L-Luc) cells were initially established through the transduction of luciferase lentivirus and selected with 25 μg/mL blasticidin S for a period of at least 2 days.

In the first in vivo experiment focused on investigating the role of METTL14 in MDS cell proliferation in vivo, the MDS-L-luc cells were transduced with the Dox-inducible shMETTL14 (shMETTL14_Tet-on). After treatment with 2 μg/mL puromycin for a duration of 4 days, a total of 2.5 × 10^6^ selected cells were injected via the tail vein into irradiated female NCG-M mice aged 8-10 weeks (GemPharmatech, China) to establish cell line-derived xenograft (CDX) models. On day 14 post transplantation, 20 mice were randomly assigned to two groups and treated with either Dox or vehicle. A total of 2 mg Dox was dissolved in water and administered by gastric lavage once a day. Five mice from each group underwent in vivo chemiluminescence imaging on day 14, 21, and 28 after receiving intraperitoneal injection of luciferin (Promega, USA). Their overall survival (OS) was also observed and documented. The remaining five mice from each group were utilized to assess the proportions of human CD45^+^ cells in bone marrow and peripheral blood on day 28 through flow cytometry analysis.

In another separate in vivo experiment aimed at investigating the impact of STM2457 on the proliferation of MDS cells in vivo, a total of 2.5 × 10^6^ MDS-L-Luc cells were injected via the tail vein into irradiated female NCG-M mice aged 8–10 weeks (GemPharmatech, China). On day 14 post transplantation, 20 mice were randomly assigned to two groups and treated with STM2457 or vehicle. STM2457 (50 mg/kg) or vehicle was delivered to the mice via intraperitoneal injection, once daily for total of two weeks (14 treatments). STM2457 was dissolved in 20% 2-hydroxyproply-beta-cyclodextrin vehicle. Five mice from each group (+ Vehicle vs + STM2457) were assigned for chemiluminescence imaging and survival monitoring. The remaining five mice in each group were utilized to assess the percentages of human CD45^+^ cells in the bone marrow on day 28.

### Statistical analysis

OS of patients was defined as the period between the date of initial diagnosis and the date of death or the last follow-up, regardless of the cause. Leukemia-free survival (LFS) was defined as the time interval from the initial diagnosis to the date of leukemic transformation or death. Patients who underwent transplantation were censored at the date of transplantation. Survival curves based on the Kaplan-Meier method were compared using the log-rank test. The independent prognostic significances of the METTL14 expression were examined by multivariate Cox regressions using a stepwise approach. Other variables included in the multivariate Cox models were age, gender, treatment therapy, and the IPSS-R. Gene mutation status with p < 0.1 in the univariate analysis was also included in the multivariate analysis.

The gene expressions of MDS patients were presented as median ± 95% confidence interval (CI), and the differences in gene expressions were assessed using the Mann-Whitney’s test. The correlation between the expressions of two genes was assessed using Pearson’s correlation test. The experimental data were analyzed using the two-tailed Student’s t-test for three independent experiments and presented as mean ± standard deviation (SD). Statistical analyses were conducted using SPSS and GraphPad Prism 9.0. P < 0.05 (two-sided) was considered statistically significant.

Other detailed materials and methods were provided in the Supplemental Methods.

## Results

### Elevated m^6^A and METTL14 level predicted unfavorable prognosis in MDS

**Fig. 1:**
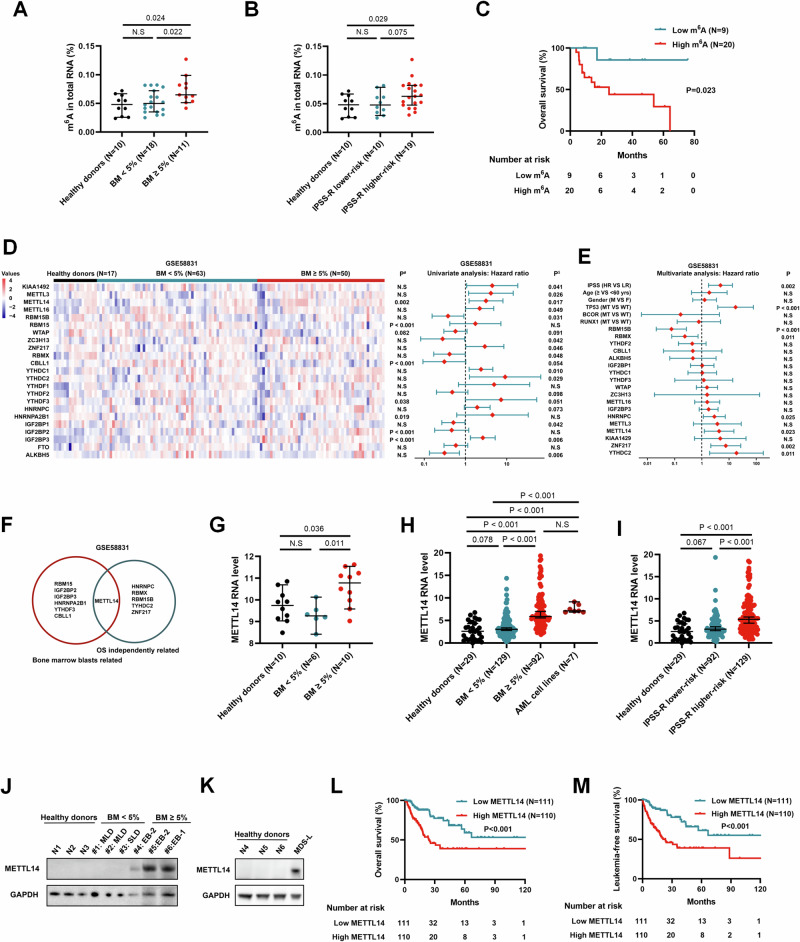
Elevated m^6^A and METTL14 level predicted unfavorable prognosis in MDS. **A** m^6^A colorimetric quantification of the global m^6^A level in bone marrow mononuclear cells (BM-MNCs) of 29 MDS patients in our center (**A**–**C**) showing higher global m^6^A level in MDS with BM ≥ 5% compared with MDS with BM < 5% and healthy donors. **B** Higher global m^6^A level in MDS with higher-risk (IPSS-R > 3.5) compared with MDS with lower-risk (IPSS-R ≤ 3.5) and healthy donors. **C** Kaplan–Meier survival analysis showing that MDS patients with high m^6^A level had shorter OS than MDS patients with low m^6^A level. **D** Gene expression and survival analyses being conducted using the publicly available MDS database (GSE58831) (**D**–**F**): the left plot showing the expression profile of 23 m^6^A regulators in bone marrow CD34^+^ cells of MDS patients stratified by the percentages of bone marrow blasts (P^#^ values based on the Kruskal-Wallis’ test); the right plot showing univariate Cox analysis (P^$^ values) of the associations of the expressions of m^6^A regulators with OS in MDS patients. **E** Multivariate Cox regression showing the independent prognostic significance of m^6^A regulators which got p < 0.1 in univariate analysis. **F** Venn diagram showing that METTL14 was the only regulator whose expression level was not only related with the percentages of bone marrow blasts but also independently associated with prognosis. **G** RNA-seq analysis of METTL14 expression in bone marrow CD34^+^ cells obtained from 16 MDS patients and 10 healthy donors in our center showing higher expression of METTL14 in MDS with BM ≥ 5% compared with MDS with BM < 5% or healthy donors. **H** q-PCR analysis of METTL14 mRNA expression in BM-MNCs obtained from 221 MDS patients in our center (**H**, **I**, **L**, **M**) showing elevated METTL14 expression in MDS patients with BM ≥ 5% compared to those with BM < 5% or healthy donors, while the expression levels of METTL14 in MDS patients with BM ≥ 5% being similar as those observed in the AML cell lines. **I** Higher METTL14 expression in MDS with higher-risk (IPSS-R > 3.5) category compared with MDS with lower-risk (IPSS-R ≤ 3.5) category or healthy donors. **J** Western blot showing METTL14 protein was highly expressed in MDS patients with BM ≥ 5%. **K** Western blot showing METTL14 protein was highly expressed in MDS cell line MDS-L. **L** Kaplan–Meier survival analysis showing that MDS patients with high METTL14 level had shorter OS than MDS patients with low METTL14 level. **M** Kaplan–Meier survival analysis showing that MDS patients with high METTL14 level had shorter LFS than MDS patients with low METTL14 level. The differences of gene expressions of MDS patients were compared using Mann-Whitney’s test or Kruskal-Wallis’s test. Kaplan–Meier survival analysis was performed using log-rank test. Error bars denoted median ± 95% CI. N.S No significance.

We measured the global m^6^A level of BM-MNCs in 29 MDS patients and 10 healthy donors. The results demonstrated that the m^6^A level was significantly higher in MDS patients with bone marrow blasts ≥ 5% than MDS patients with blasts < 5% and healthy donors (Fig. 1A). Additionally, higher m^6^A level was observed in higher-risk MDS patients (IPSS-R scores > 3.5) according to the IPSS-R, while lower-risk MDS patients (IPSS-R scores ≤ 3.5) did not exhibit a noticeable difference in m^6^A level compared to healthy donors (Fig. 1B). Moreover, patients with high m^6^A level had an obviously shorter OS in comparison to those with low m^6^A level (Fig. 1C).

To identify the key m^6^A regulator accounted for the dysregulated m^6^A modification in MDS, the expressions of 23 m^6^A regulators in bone marrow CD34^+^ cells were analyzed based on a publicly available gene expression profile of MDS cohort (GSE58831). The results revealed that the expression levels of METTL14 and other six m^6^A regulators were notably different among the three groups divided by the percentages of bone marrow blasts (healthy donors, MDS patients with blasts < 5%, and MDS patients with blasts ≥ 5%) (Fig. 1D). Further survival analyses identified that METTL14, HNRNPC, RBMX, RBM15B, YTHDC2, and ZNF217 were independently associated with prognosis (Fig. 1D, E), among them, METTL14 was the sole regulator whose expression level was not only correlated with the bone marrow blasts but also independently related with survival (Fig. 1F).

We then verified the expression of METTL14 in MDS samples from our own cohorts. As expected, METTL14 expression in CD34^+^ cells of BM-MNCs from MDS patients with blasts ≥ 5% was higher than that from healthy donors (Fig. 1G). Consistently, METTL14 mRNA was highly expressed in BM-MNCs of MDS cases with blasts ≥ 5%, and was upregulated as the IPSS-R risk increased (Fig. 1H, I; Supplementary Fig. 1). Accordingly, we observed elevated protein level of METTL14 in MDS patients with blasts ≥ 5% (Fig. 1J) and MDS cell line MDS-L (Fig. 1K). Moreover, higher METTL14 mRNA level in BM-MNCs of MDS patients was independently associated with shorter survival (P = 0.025, HR = 1.932, 95%CI: 1.086–3.440; Fig. 1L; Supplementary Table 4) and faster leukemic transformation (P = 0.006, HR = 2.320, 95%CI: 1.271–4.233; Fig. 1M; Supplementary Table 5), after adjustment of the IPSS-R, age, gender, mutation status, and treatment therapy. Collectively, m^6^A level and METTL14 expression were elevated in MDS with bone marrow blasts ≥ 5%, and significantly associated with disease risks and clinical outcomes, suggesting that METTL14 may play crucial roles in the pathogenesis of MDS.

### METTL14 promoted MDS cell proliferation in vitro and in vivo

**Fig. 2:**
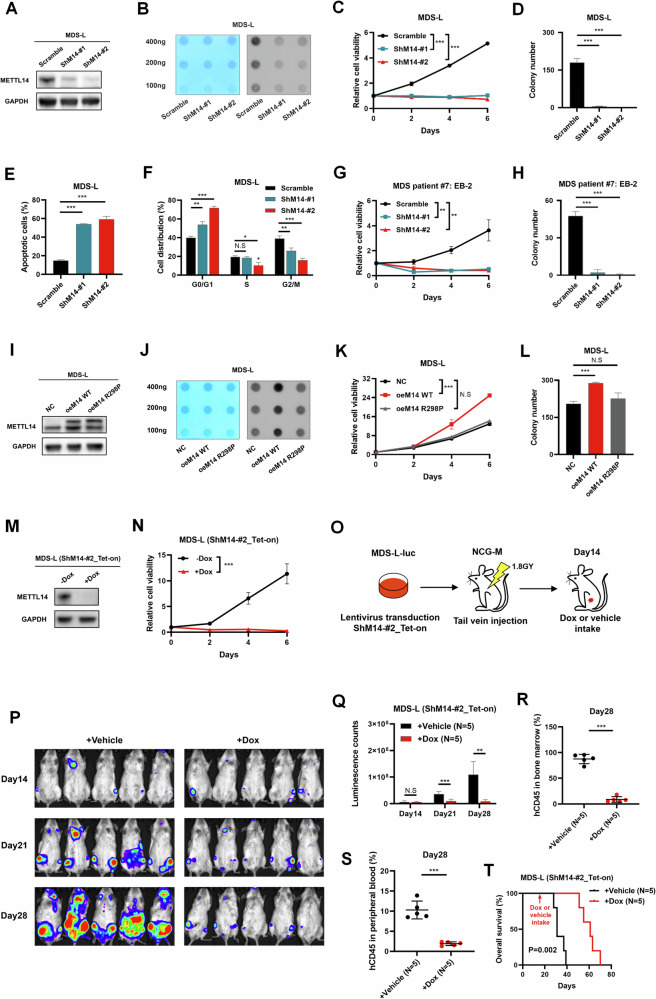
METTL14 promotes MDS cell proliferation in vitro and in vivo. **A** Western blot showing the knockdown effect of METTL14 in METTL14-knockdown MDS-L cells which generated through lentivirus. **B** Dot blot assay showing METTL14 knockdown led to obviously decreased m^6^A modification of MDS-L cells. **C** CellTiter-Lumi^TM^ assay showing that knockdown of METTL14 significantly inhibited the cell proliferation of MDS-L cells. ***P < 0.001. **D** The colony formation assay showing that knockdown of METTL14 markedly inhibited the colony formation of MDS-L cells. ***P < 0.001. **E** Flow cytometry showing that knockdown of METTL14 obviously increased the apoptosis of MDS-L cells. ***P < 0.001. **F** Flow cytometry showing that knockdown of METTL14 blocked the cell cycle in G0/G1 phase of MDS-L cells. ***P < 0.001; **P = 0.003 (left), 0.005 (right); *P = 0.014. **G** CellTiter-Lumi^TM^ assay showing that knockdown of METTL14 markedly inhibited the cell proliferation of CD34^+^ MDS blasts from a patient with EB-2. **P = 0.003 (left), 0.003 (right). **H** The colony formation assay showing that knockdown of METTL14 significantly inhibited the colony formation of CD34^+^ MDS blasts from a patient with EB-2. ***P < 0.001. **I** Western blot showing the overexpression effect of METTL14 in METTL14 WT and METTL14 R298P-overexpressing MDS-L cells. **J** Dot blot assay showing that overexpression of METTL14 WT, but not METTL14 R298P, increased m^6^A levels of MDS-L cells. **K** CellTiter-Lumi^TM^ assay showing that overexpression of METTL14 WT, but not METTL14 R298P, significantly enhanced the cell proliferation of MDS-L cells. ***P < 0.001. **L** The colony formation assay showing that overexpression of METTL14 WT, but not METTL14 R298P, significantly enhanced the colony formation of MDS-L cells. ***P < 0.001. **M** Western blot showing the knockdown effect of METTL14 in MDS-L cells expressing Dox-inducible shMETTL14 (shMETTL14_Tet-on) after 2 days of Dox treatment. **N** CellTiter-Lumi^TM^ assay showing that knockdown of METTL14 by a Dox-inducible shMETTL14 significantly inhibited the cell proliferation of MDS-L cells in vitro. ***P < 0.001. **O** In vivo experimental scheme for (**O**–**T**): briefly, NCG-M mice xenografted with the MDS-L-luc cells transduced with Dox-inducible shMETTL14 subjected to the Dox treatment or the vehicle. (**P**, **Q**) Chemiluminescence imaging (**P**) and its luminescence counts (**Q**) showing a noticeable suppression of MDS-L-luc cells engraftment in mice following Dox-induced knockdown of METTL14. ***P < 0.001; **P = 0.002. **R**, **S** Flow cytometry showing that Dox-induced knockdown of METTL14  in vivo remarkably decreased the percentages of human CD45^+^ cells in bone marrow (**R**) and peripheral blood (**S**) of mice. ***P < 0.001. **T** Kaplan–Meier survival analysis showing that Dox-induced knockdown of METTL14 in vivo prolonged the OS of mice. The experimental data were analyzed with Student’s t-test. Kaplan–Meier survival analysis was performed using log-rank test. Error bars denoted mean ± SD of three independent experiments. N.S No significance.

We next explored the biological function of METTL14 in MDS cells. We firstly knocked down METTL14 in MDS-L cells (Fig. 2A) and found that suppression of METTL14 markedly reduced the m^6^A level in MDS-L cells (Fig. 2B; Supplementary Fig. 2). Moreover, METTL14 knockdown significantly impeded cell growth (Fig. 2C) and colony formation of MDS-L cells (Fig. 2D), while promoted cell apoptosis (Fig. 2E) and arrested the cell cycle in the G0/G1 phase (Fig. 2F). Similarly, the knockdown of METTL14 resulted in the inhibition of cell growth and colony formation of CD34^+^ MDS cells (Fig. 2G, H). In contrast, enforced expression of METTL14 wild type (WT), but not METTL14 R298P (a catalytically inactive mutant), increased m^6^A level (Fig. 2I, J). Moreover, the overexpression of METTL14 WT, but not METTL14 R298P, significantly augmented cell proliferation (Fig. 2K) and colony formation of MDS-L cells (Fig. 2L).

Furthermore, we explored the role of METTL14 in MDS cell proliferation in vivo. A Dox-inducible shMETTL14 was constructed and used to knock down METTL14 in MDS-L cells (Fig. 2M), resulting in a significant inhibition of in vitro proliferation after Dox induction (shMETTL14-ON) (Fig. 2N). Subsequently, MDS-L-Luc cells transduced with the Dox-inducible shMETTL14 were intravenously injected into irradiated mice (Fig. 2O). Chemiluminescence imaging revealed a significant suppression of engrafted MDS-L cells following METTL14 knockdown induced by Dox (Fig. 2P, Q). Additionally, the proportions of MDS-L cells in both bone marrow and peripheral blood were significantly reduced upon METTL14 knockdown as well (Fig. 2R, S). As expected, the knockdown of METTL14 in MDS-L cells resulted in prolonged survival of the recipients (Fig. 2T). Altogether, these findings suggested that METTL14 played an essential role in the growth of MDS cells both in vitro and in vivo.

### Identification of potential downstream targets of METTL14 in MDS

**Fig. 3:**
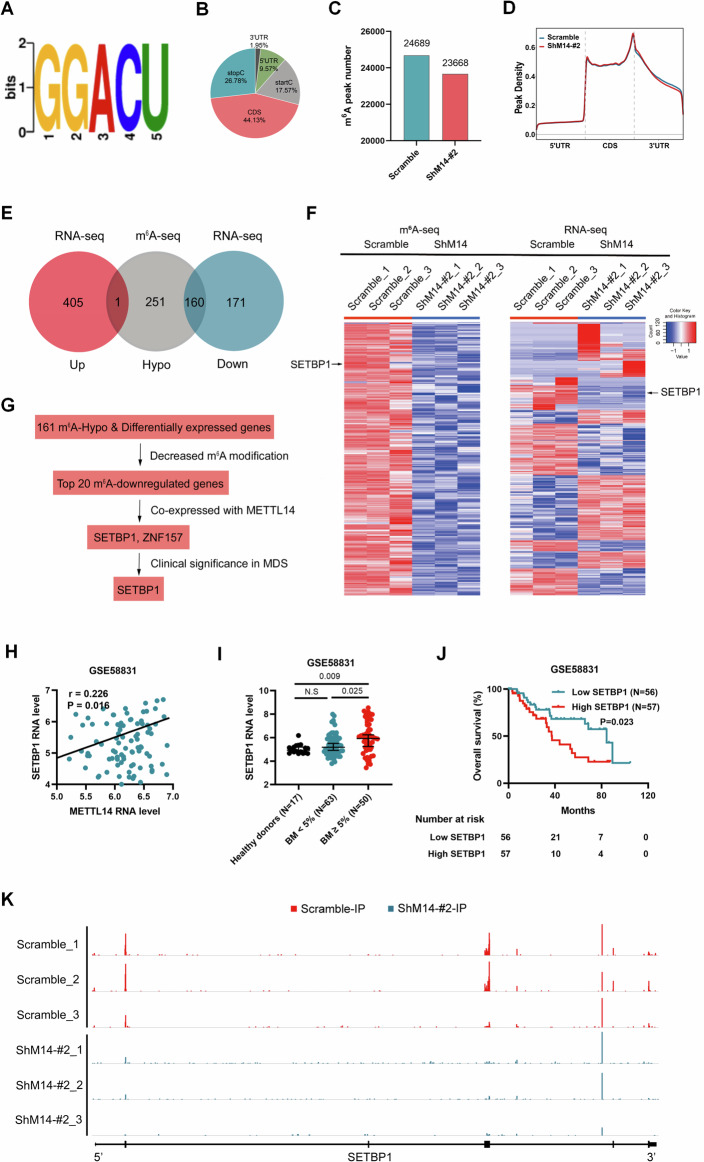
Identification of potential downstream targets of METTL14 in MDS by m^6^A-seq and RNA-seq. **A** m^6^A-seq identifying GGACU as the m^6^A consensus sequence motif in MDS-L cells. **B** The distribution of m^6^A peaks in MDS-L cells indicated by m^6^A-seq. **C** m^6^A-seq showing the numbers of m^6^A peaks in METTL14-knockdown MDS-L cells and the control MDS-L cells. **D** The difference of m^6^A peak density in METTL14-knockdown MDS-L cells and the control MDS-L cells indicated by m^6^A-seq. **E** Venn diagram showing the m^6^A-hypo genes indicated by m^6^A-seq (fold-change ≥ 2 or ≤ -2; P values < 0.00001) and differentially expressed genes indicated by RNA-seq (fold-change ≥ 2 or ≤ -2; P values < 0.05) upon METTL14 knockdown. **F** Heap maps showing the genes with decreased m^6^A level indicated by m^6^A-seq and differentially expressed genes indicated by RNA-seq upon METTL14 knockdown. **G** The specific steps for which SETBP1 was selected as the potential key target of METTL14. **H** Pearson’s correlation test showing positive correlation between METTL14 and SETBP1 in the GSE58831 MDS cohort. **I** Higher expression of SETBP1 in MDS with BM ≥ 5% compared with MDS with BM < 5% or healthy donors in the GSE58831 MDS cohort. **J** Kaplan–Meier survival analysis showing that MDS patients with high SETBP1 level had shorter OS than MDS patients with low SETBP1 level in the GSE58831 MDS cohort. **K** Visualization of m^6^A modification of SETBP1 mRNA in METTL14-knockdown MDS-L cells and the control MDS-L cells by IGV. N.S No significance.

The analysis of m^6^A-seq data revealed that the consensus sequence for m^6^A modification in MDS-L cells was GGACU (Fig. 3A), previously recognized as the common motif of m^6^A modification [14, 25]. The m^6^A modification in MDS-L cells was predominantly located in the coding sequence (CDS) regions (44.13%), followed by the stop codon sites (26.78%), start codon sites (17.57%), 5’-UTR regions (9.57%), and 3’-UTR regions (1.95%) (Fig. 3B). In the control MDS-L cells, a total of 24689 m^6^A peaks were detected, whereas 23668 m^6^A peaks were observed in the METTL14-knockdown MDS-L cells (Fig. 3C). Notably, the m^6^A peak density in 3’-UTR regions of the METTL14-knockdown MDS-L cells was lower compared with that in the control MDS-L cells (Fig. 3D).

We identified 412 mRNA transcripts with a significant decrease in m^6^A modification (referred to as m^6^A-hypo) (Fig. 3E), and 413 mRNA transcripts with an obvious increase in m^6^A modification (referred to as m^6^A-hyper) upon METTL14 knockdown (fold-change ≥ 2 or ≤ -2; P values < 0.00001). Meanwhile, METTL14 knockdown led to downregulation of 331 mRNA transcripts and upregulation of 406 mRNA transcripts (fold-change ≥ 2 or ≤ -2; P values < 0.05; Fig. 3E). Considering the m^6^A methylation activity of METTL14, we hypothesized that decreased m^6^A modification might be directly caused by METTL14 knockdown, while upregulated m^6^A level might be an indirect effect, thus our study focused on the m^6^A-hypo genes upon METTL14 knockdown. The genes with decreased m^6^A level or differential expression were presented as heatmaps respectively (Fig. 3F), which included the candidate target (black arrow indicated) discussed as follows. Through an integrative analysis of m^6^A-seq and RNA-seq data, we found that the expressions of 160 m^6^A-hypo mRNA transcripts were simultaneously downregulated, while the expression of one m^6^A-hypo mRNA transcript was upregulated (Fig. 3E). To further narrow down the target genes of METTL14 in MDS (Fig. 3G), the top 20 m^6^A-downregulated genes were screened out from the 161 m^6^A-hypo and differentially expressed genes as the potential candidate genes. Subsequently, the correlations between these potential target genes and METTL14 were examined using the expression data from GSE58831. The results revealed significant relationships between the expressions of SETBP1 and ZNF157 with METTL14 (Fig. 3H; Supplementary Table 6). Finally, gene expression and survival analyses were conducted and we found that the expression of SETBP1, but not ZNF157, had clinical significance according to the GSE58831 cohort (Fig. 3I, J; Supplementary Fig. 3). Specifically, SETBP1 was highly expressed in MDS with blasts ≥ 5%, and high expression of SETBP1 was associated with adverse prognosis. The reduction in m^6^A modification in SETBP1 transcripts caused by METTL14 knockdown was then confirmed and visualized using IGV (Fig. 3K). Thus, we identified SETBP1 as a potential critical target of METTL14 in MDS.

### METTL14 stabilized SETBP1 mRNA through METTL3-METTL14 complex-mediated m^6^A modification in MDS

**Fig. 4:**
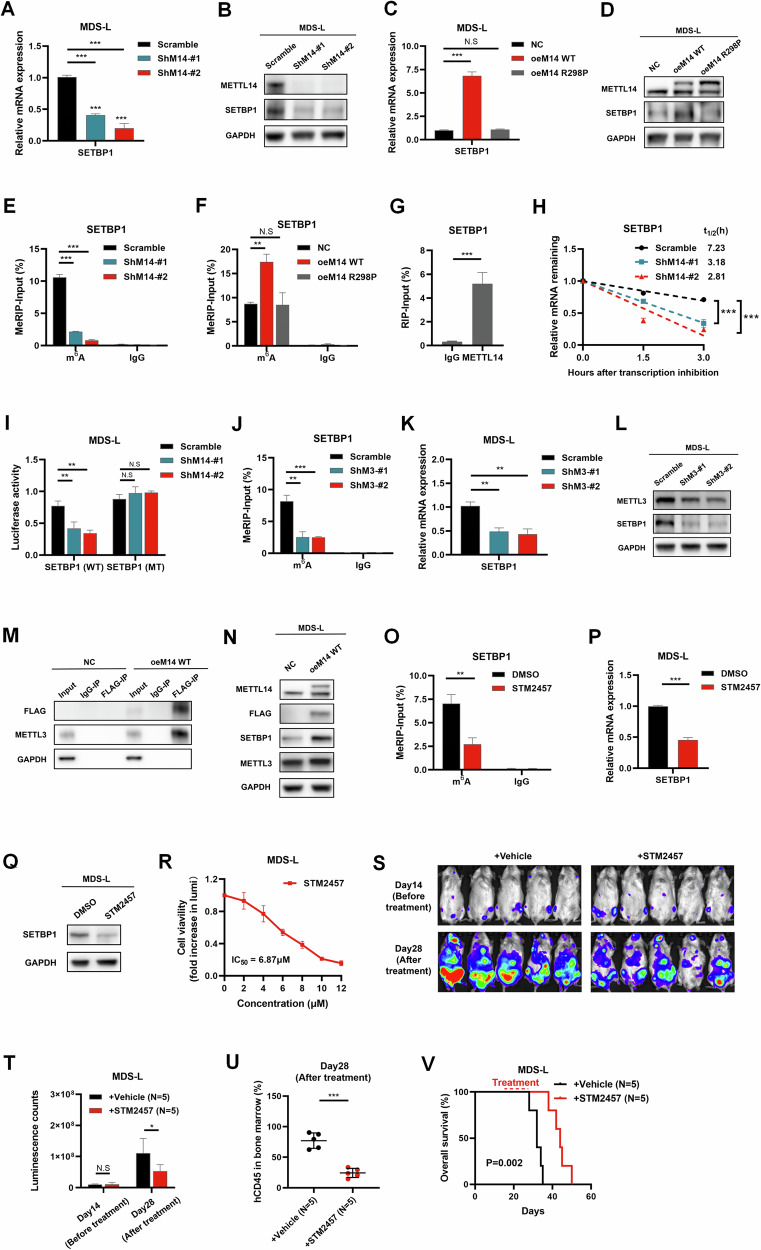
SETBP1 was a direct target of METTL14-mediated m^6^A modification in MDS. **A** Obvious decrease of SETBP1 mRNA in MDS-L cells with METTL14 knockdown indicated by q-PCR analysis. ***P < 0.001. **B** Western blot showing METTL14 knockdown led to significant decrease of SETBP1 protein in MDS-L cells. **C** Noticeable increase of SETBP1 mRNA in MDS-L cells with overexpression of METTL14 WT, but not the METTL14 R298P, indicated by q-PCR analysis. ***P < 0.001. **D** Western blot showing forced expression of METTL14 WT, but not the METTL14 R298P, increased SETBP1 protein. **E** MeRIP-qPCR showing a remarkable reduction of m^6^A modification in specific regions of SETBP1 mRNA transcripts upon METTL14 knockdown. ***P < 0.001. **F** MeRIP-qPCR showing an increase of m^6^A modification in MDS-L cells with overexpression of METTL14 WT, but not the METTL14 R298P. **P = 0.001. **G** RIP-qPCR showing METTL14 protein bound to SETBP1 mRNA in MDS-L cells. ***P < 0.001. **H** RNA stability assays showing that METTL14 knockdown decreased the half-life of SETBP1 mRNA. ***P < 0.001. **I** Dual-luciferase assays of SETBP1 WT 3’-UTR-reporter showing the decreased activity of luciferase upon METTL14 knockdown, while METTL14 knockdown had no effect on SETBP1 3’-UTR-reporter with mutated m^6^A sites. **P = 0.008 (left), 0.001 (right). **J** MeRIP-qPCR showing a significant reduction of m^6^A modification in specific regions of SETBP1 mRNA transcripts upon METTL3 knockdown. **P = 0.002; ***P < 0.001. **K** Significant decrease of SETBP1 mRNA in MDS-L cells with METTL3 knockdown indicated by q-PCR analysis. **P = 0.002 (left), 0.001 (right). **L** Western blot showing METTL3 knockdown led to significant decrease of SETBP1 protein in MDS-L cells. **M** Co-IP experiments showing the overexpression of METTL14 led to an increase in the binding with METTL3. **N** Western blot showing the overexpression of METTL14 did not alter the overexpression of METTL3. **O** MeRIP-qPCR showing a remarkable reduction of m^6^A modification in specific regions of SETBP1 mRNA transcripts treatment with STM2457 (10 μM) for 48 hours. **P = 0.003. **P** Obvious reduce of SETBP1 mRNA in MDS-L cells treated with STM2457 (10 μM) for 48 hours indicated by q-PCR analysis. ***P < 0.001. **Q** Western blot showing treatment with STM2457 (10 μM) for 48 hours led to significant decrease of SETBP1 protein in MDS-L cells. **R** CellTiter-Lumi^TM^ assay showing that treatment with STM2457 for 48 hours resulting in concentration-dependent inhibition of cell viability of MDS-L cells, with an IC_50_ of 6.87 μM. **S**, **T** Chemiluminescence imaging (S) and its luminescence counts (**T**) showing a noticeable suppression of MDS-L-luc cells engraftment in mice after treatment of STM2457 (50 mg/kg). *P = 0.034. **U** Flow cytometry showing that treatment of STM2457 (50 mg/kg) remarkably decreased the percentages of human CD45^+^ cells in bone marrow of mice. ***P < 0.001. **V** Kaplan–Meier survival analysis showing that treatment of STM2457 (50 mg/kg) in vivo prolonged the OS of mice. The experimental data were analyzed with Student’s t-test. Error bars denoted mean ± SD of three independent experiments. *P < 0.05; **P < 0.01; ***P < 0.001; N.S No significance.

We found that METTL14 knockdown led to noticeably decreased SETBP1 mRNA and protein expressions in MDS-L cells (Fig. 4A, B). On the contrary, the forced expression of METTL14 WT, rather than METTL14 R298P, resulted in an increase in both mRNA and protein levels of SETBP1 (Fig. 4C, D). These findings suggested that METTL14 positively regulated the expression of SETBP1.

Further, we found that m^6^A modification of SETBP1 mRNA was markedly decreased upon METTL14 knockdown (Fig. 4E). Conversely, the forced expression of METTL14 WT, but not the METTL14 R298P, enhanced the m^6^A abundance of SETBP1 mRNA in MDS-L cells (Fig. 4F). These results indicated that METTL14 played a positive regulatory role in the m^6^A modification of SETBP1 mRNA. Additionally, SETBP1 mRNA was significantly enriched by anti-METTL14 antibody in MDS-L cells (Fig. 4G), which suggesting that METTL14 protein bound to SETBP1 mRNA in MDS-L cells. m^6^A modification was reported to modulate gene expression by regulating mRNA stability [26–28]. To further explore whether METTL14 affect mRNA stability of SETBP1, RNA stability assay was conducted. The results showed that METTL14 knockdown significantly decreased the half-life of SETBP1 mRNA (Fig. 4H), which indicating that METTL14 enhanced SETBP1 mRNA stability. In order to determine whether METTL14 regulated SETBP1 mRNA stability relying on m^6^A modification, dual-luciferase assay was performed. The results revealed that the activity of luciferase of SETBP1-3’UTR-WT reporter decreased upon METTL14 knockdown, while mutations at m^6^A motif sites in SETBP1-3’UTR-WT reporter abolished this effect (Fig. 4I), suggesting an m^6^A-dependent regulation. Collectively, our data indicated that METTL14 promoted SETBP1 mRNA stability via increasing m^6^A modification of SETBP1 transcripts.

As indicated in the literature, METTL14 regulated the deposition of m^6^A modification by forming a heterodimer complex with METTL3 [25, 29, 30]. Thus, we next investigated the role of METTL3-METL14 complex in the regulation of m^6^A modification of SETBP1 mRNA. A marked reduction in m^6^A modification of SETBP1 mRNA was observed in METTL3-knockdown cells (Fig. 4J), with a consequently decreased both of SETBP1 mRNA and protein (Fig. 4K, L). These findings suggested that METTL3 was involved in m^6^A modification of SETBP1 mRNA in MDS cells. Moreover, overexpression of METTL14 promoted METTL3-METTL14 complex formation as evidenced by additional METTL3 protein pulled down by FLAG-tagged METTL14 (Fig. 4M), while did not alter the mRNA and protein levels of METTL3 (Fig. 4N; Supplementary Fig. 4). The small molecule STM2457 has been reported to specifically inhibit the catalytic activity of METTL3-METTL14 complex [19]. MDS-L cells treated with STM2457 exhibited a reduction in m^6^A modification in SETBP1 transcripts (Fig. 4O), followed by decreased levels of SETBP1 mRNA and protein (Fig. 4P, Q). These data suggested that METTL14 enhanced the m^6^A modification of SETBP1 mRNA through forming a complex with METTL3. Collectively, our data suggested that METTL14 facilitated the m^6^A modification of SETBP1 mRNA by formation of METTL3-METTL14 complex.

Furthermore, we proceeded to investigate the effects of STM2457 on the growth of MDS cells. Treatment with STM2457 resulted in concentration-dependent inhibition of cell viability of MDS-L cells, with an IC_50_ of 6.87 μM (Fig. 4R). We also evaluated the therapeutic efficacy of STM2457 on MDS in vivo. The results demonstrated that STM2457 distinctly inhibited MDS-L cell engraftment (Fig. 4S–U) and extended the survival of the recipient mice (Fig. 4V). Altogether, our data supported that the METTL3-METTL14 heterodimer was essential for the m^6^A modification of SETBP1 mRNA, highlighting its potential as a promising target for MDS therapy.

### SETBP1 was elevated in MDS with blasts ≥ 5%, and promoted MDS cell proliferation

**Fig. 5:**
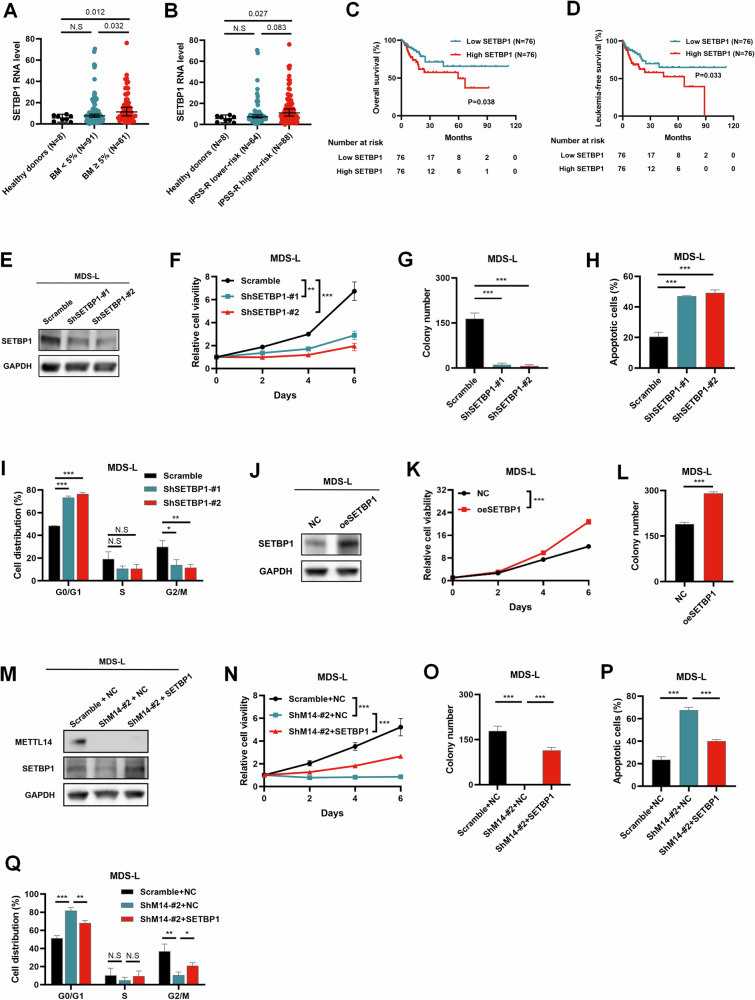
SETBP1 acted as an oncogene in MDS, METTL14-m^6^A-SETBP1 regulated PI3K-AKT signaling pathway in MDS. **A** q-PCR analysis of SETBP1 in bone marrow mononuclear cells (BM-MNCs) of MDS patients in our center (**A**–**D**) showing higher expression of SETBP1 detected in MDS with BM ≥ 5% compared with MDS with BM < 5% or healthy donors. **B** SETBP1 expression in MDS with higher-risk (IPSS-R > 3.5), MDS with lower-risk (IPSS-R ≤ 3.5), and healthy donors. **C** Kaplan–Meier survival analysis showing that MDS patients with high SETBP1 level had shorter OS than MDS patients with low SETBP1 level. **D** Kaplan–Meier survival analysis of MDS patients in our center showing that MDS patients with high SETBP1 level had shorter LFS than MDS patients with low SETBP1 level. **E** Western blot showing the knockdown effect of SETBP1 in SETBP1-knockdown MDS-L cells. **F** CellTiter-Lumi^TM^ assay showing that knockdown of SETBP1 significantly inhibited the cell proliferation. **P = 0.002; ***P < 0.001. **G** The colony formation assay showing that knockdown of SETBP1 obviously inhibited the colony-formation ability. ***P < 0.001. **H** Flow cytometry showing that knockdown of SETBP1 significantly increased the apoptosis of MDS-L cells. ***P < 0.001. **I** Flow cytometry showing that knockdown of SETBP1 blocked the cell cycle in G0/G1 phase. ***P < 0.001; *P = 0.017; **P = 0.006. **J** Western blot showing the overexpression effect of SETBP1 in SETBP1-overexpressing MDS-L cells. **K** CellTiter-Lumi^TM^ assay showing that overexpression of SETBP1 notably promoted the cell proliferation. ***P < 0.001. **L** The colony formation assay showing that overexpression of SETBP1 significantly enhanced the colony-formation ability. ***P < 0.001. **M** Western blot showing the protein levels of METTL14 and SETBP1 in the MDS-L cells transduced with indicated lentiviruses. **N** CellTiter-Lumi^TM^ assay showing that restoration of SETBP1 expression partly rescued the inhibition of cell proliferation caused by METTL14 knockdown in MDS-L cells. ***P < 0.001. **O** The colony formation assay showing that restoration of SETBP1 expression partly rescued the inhibition of colony formation ability caused by METTL14 knockdown in MDS-L cells. ***P < 0.001. **P** Flow cytometry showing that restoration of SETBP1 expression partly rescued the increase of the apoptosis of MDS-L cells caused by METTL14 knockdown. ***P < 0.001. **Q** Flow cytometry showing that restoration of SETBP1 expression partly rescued the cell cycle arrest caused by knockdown of METTL14. ***P < 0.001; **P = 0.005 (left), 0.006 (right);*P = 0.020. The differences of gene expressions of MDS patients were compared using Mann-Whitney’s t-test. Error bars of gene expression denoted median ± 95% CI. Kaplan–Meier survival analysis was performed using log-rank test. The experimental data were analyzed with Student’s t-test. Error bars of experimental data denoted mean ± SD of three independent experiments. *P < 0.05; **P < 0.01; ***P < 0.001; N.S No significance.

In line with the GSE58831 cohort, the data of our own center showed SETBP1 mRNA was elevated in MDS patients with blasts ≥ 5% (Fig. 5A) compared with healthy donors, and parallel with disease risk (Fig. 5B). Additionally, our cohort demonstrated that MDS patients with high expression of SETBP1 had shorter survival and faster leukemic transformation than those with low SETBP1 expression (Fig. 5C, D).

To further explore the biological functions of SETBP1 in MDS, we firstly knocked down SETBP1 in MDS-L cells (Fig. 5E) and found that knockdown of SETBP1 significantly inhibited cell growth (Fig. 5F) and colony formation (Fig. 5G), but increased cell apoptosis (Fig. 5H), and arrested the cell cycle at the G0/G1 phase (Fig. 5I). In contrast, overexpression of SETBP1 (Fig. 5J) noticeably promoted cell proliferation (Fig. 5K) and colony formation of MDS-L cells (Fig. 5L).

In order to determine whether METTL14 promoted MDS cell proliferation via SETBP1, we restored SETBP1 expression in METTL14-knockdown MDS-L cells (Fig. 5M). Ectopic expression of SETBP1 partially reversed the inhibitory effects of METTL14 deficiency on cell growth (Fig. 5N) and colony-formation ability of MDS-L cells (Fig. 5O). Moreover, the restoration of SETBP1 expression also partially rescued the cell apoptosis (Fig. 5P) and cell arrest caused by METTL14 deficiency (Fig. 5Q). Taken together, these data provided evidences that SETBP1 was elevated in MDS with blasts ≥ 5% and higher-risk category, and promoted MDS cell proliferation, which contributing to the functions of METTL14 in MDS.

### METTL14-m^6^A-SETBP1 regulated PI3K-AKT signaling pathway in MDS

**Fig. 6:**
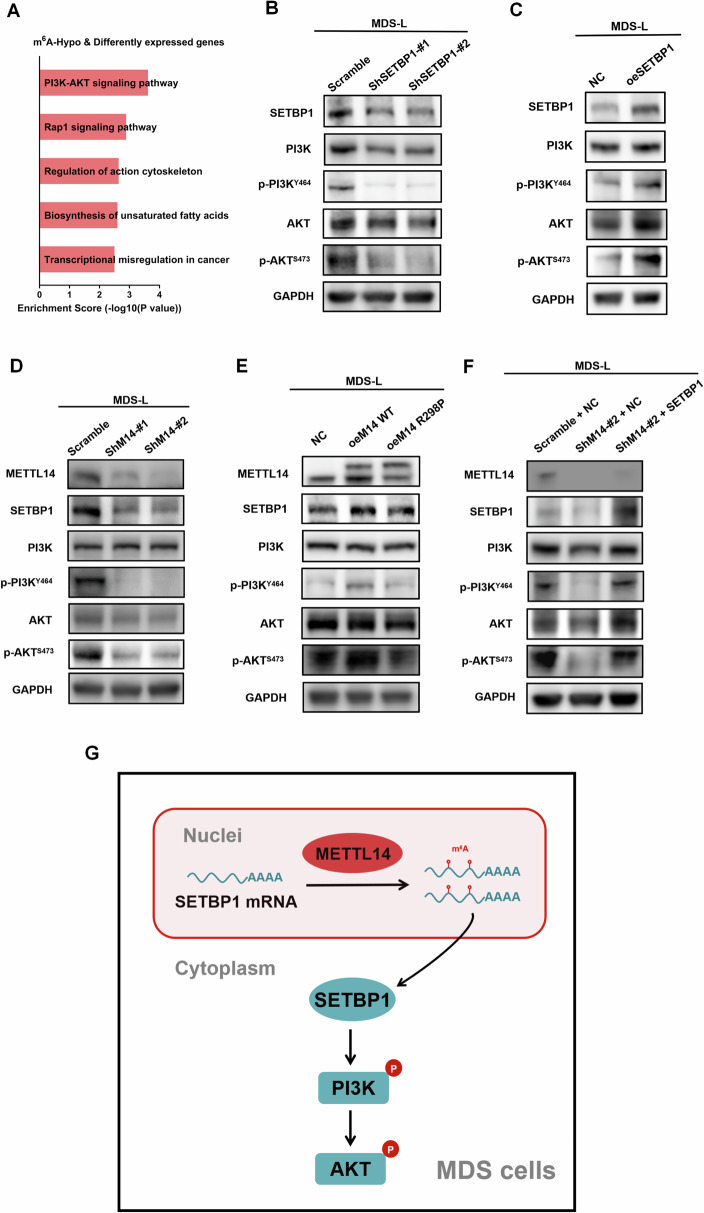
METTL14-m^6^A-SETBP1 axis regulated PI3K-AKT pathway in MDS. **A** KEGG pathway analysis of m^6^A-hypo and differentially expressed genes upon METTL14 knockdown. **B** Western blot showing the knockdown of SETBP1 in MDS-L cells led to downregulation of PI3K-AKT signaling pathway. **C** Western blot showing the overexpression of SETBP1 in MDS-L cells led to upregulation of PI3K-AKT signaling pathway. **D** Western blot showing the knockdown of METTL14 in MDS-L cells led to reduction of SETBP1 expression and downregulation of PI3K-AKT signaling pathway. **E** Western blot showing the overexpression of METTL14-WT, but not METTL14-R298P, in MDS-L cells led to promotion of SETBP1 expression and activation of PI3K-AKT signaling pathway. **F** Western blot showing that the downregulation of PI3K-AKT signaling pathway by METTL14 inhibition was rescued by SETBP1 restoration. **G** Proposed model depicting the regulation and the role of METTL14 in MDS progression. In MDS cells, METTL14 enhancing SETBP1 mRNA stability and promoting the expression of SETBP1 via increasing m^6^A abundance of SETBP1 transcripts; PI3K-AKT signaling pathway being the downstream of METTL14-m^6^A-SETBP1 to induce MDS tumorigenesis.

Through KEGG analysis of the m^6^A-hypo and differentially expressed genes after METTL14 knockdown which indicated by m^6^A-seq and RNA-seq data, the PI3K-AKT pathway was identified as the potential signaling pathway of METTL14 in MDS (Fig. 6A). According to the literature, SETBP1 played its oncogenic role in AML through protecting oncogene SET from protease cleavage, consequently leading to the inhibition of PP2A [31, 32]. PP2A, as a crucial tumor suppressor gene, had been shown to deactivate the PI3K-AKT signaling pathway in cancer cells [33–36]. In light of the above, we hypothesized that METTL14-m^6^A-SETBP1 axis had a positive regulation on PI3K-AKT pathway in MDS cells. As expected, we found that SETBP1 inhibition downregulated the PI3K-AKT pathway (Fig. 6B), while overexpression of SETBP1 activated the PI3K-AKT signaling pathway in MDS-L cells (Fig. 6C). Moreover, knockdown of METTL14 in MDS-L cells not only reduced SETBP1 expression but also suppressed the PI3K-AKT signaling pathway (Fig. 6D). In contrast, overexpression of METTL14 WT, but not METTL14 R298P, led to promotion of SETBP1 expression and activation of PI3K-AKT signaling pathway (Fig. 6E). More importantly, we found that the downregulation of PI3K-AKT signaling pathway by METTL14 inhibition could be rescued by SETBP1 restoration (Fig. 6F). Collectively, these data suggested that METTL14-m^6^A-SETBP1 axis promoted MDS cell proliferation by activation of PI3K-AKT signaling pathway (Fig. 6G).

## Discussion

Studies have shown the significant involvement of m^6^A modification and m^6^A regulators in the pathogenesis of various malignant tumors [15, 37–40]. However, the roles of m^6^A modification and m^6^A regulators in MDS were unclear. The present study revealed that m^6^A level and METTL14 expression were increased in MDS patients with blasts ≥ 5%. In addition, elevations of m^6^A modification and METTL14 mRNA were associated with high disease risks and unfavorable clinical outcomes. METTL14 facilitated the proliferation of MDS cells by promoting the m^6^A modification of SETBP1 mRNA through the formation of the METTL3-METTL14 complex, leading to enhanced stability of SETBP1 mRNA and subsequent activation of the PI3K-AKT signaling pathway. These findings revealed the crucial contributions of METTL14 and METTL3-METTL14 complex-mediated m^6^A modification in the pathogenesis of MDS.

METTL14 functions as an important m^6^A methyltransferase that regulates the deposition of m^6^A modifications through formation of heterodimers with METTL3 [25, 29, 30]. Accumulating evidence indicated that METTL14 played pivotal but distinct roles in various cancers [16, 40–44]. For instance, in AML, METTL14 promoted leukemogenesis by modulating the expressions of MYB and MYC through m^6^A modification [16]. In osteosarcoma, METTL14 facilitated tumor progression by upregulating the m^6^A modification of MN1 mRNA [41]. Conversely, in liver cancer, METTL14 impeded the invasion and metastasis of cancer cells by regulating miR-126 [45]. In bladder cancer, METTL14 suppressed the development and progression of cancer cells by regulating Notch1 [46]. Consistent with the role of METTL14 in AML, our study demonstrates that METTL14 also contributed to the pathogenesis of MDS by enhancing the cell proliferation and colony-forming ability of MDS cells. Furthermore, our findings indicated that the small molecule STM2457 targeting METTL3-METTL14 complex exerted similar inhibitory effects on MDS cells, both in vitro and in vivo, as it does on AML cells [19]. These results underscore the critical role of METTL14 in the survival and growth of MDS cells, highlighting the potential therapeutic value of targeting the METTL3-METTL14 complex-mediated m^6^A modification in MDS.

However, unlike the downstream mechanism of METTL14 in AML, our research demonstrated that SETBP1 was the direct downstream target gene of METTL14 in MDS. METTL14 promoted MDS development by enhancing the m^6^A modification of SETBP1 mRNA through formation of METTL3-METTL14 complex, leading to increased stability and expression of SETBP1 mRNA. Furthermore, the restoration of SETBP1 expression partially alleviated the suppression of cell proliferation induced by METTL14 knockdown, implying that the oncogenic effects of METTL14 in MDS were partially reliant on its regulation of SETBP1 expression. SETBP1 was reported to be highly expressed in a subset of AML [20] and CML patients with blast crisis [23], and promoted leukemogenesis by inhibiting tumor suppressor gene PP2A [31]. Besides, missense mutations in SETBP1 were detected in some AML and MDS patients, and associated with worse prognosis and faster disease progression [47, 48]. According to the literature, both the overexpression of SETBP1-WT and the presence of missense mutations would activate SETBP1 to promote tumorigenesis in myeloid leukemia [49, 50]. In the present study, we found that SETBP1 was highly expressed in MDS patients with blasts ≥5%, and this overexpression was found to be associated with poor prognosis and rapid leukemic transformation. Further experiments revealed that SETBP1 exerted an oncogenic role by facilitating the MDS cell proliferation and enhancing the colony formation ability.

Furthermore, PI3K-AKT signaling pathway was confirmed as the downstream signaling pathway of the METTL14-m^6^A-SETBP1 axis. The regulation of METTL14 on the PI3K-AKT signaling pathway was observed in several malignant tumors such as liver cancer [39] and gastric cancer [42], which was also validated in our study. SETBP1 was reported to play its oncogenic role by inhibiting PP2A in AML [31]. Notably, PP2A had been shown to restrict the PI3K-AKT signaling pathway in certain cancer cells [33–36]. In our study, we observed the regulations of SETBP1 on the PI3K-AKT signaling pathway in MDS cells, and found that METTL14 activated the PI3K-AKT signaling pathway by upregulating SETBP1 expression.

## Conclusion

In summary, this research has illustrated the significant oncogenic function of METTL14 in the development of MDS. METTL14 enhances m^6^A modification of SETBP1 mRNA through the formation of METTL3-METTL14 complex, leading to increased stability of SETBP1 mRNA and activation of the PI3K-AKT signaling pathway, ultimately facilitating MDS cell proliferation. Overall, our findings suggested that targeting the METTL3-METTL14 complex-mediated m^6^A modification could provide a promising therapeutic approach for MDS.

## Supplementary information

Supplemental Methods

Supplementary Table 1

Supplementary Table 2-6

Supplementary Figure 1-4

## Data Availability

The detailed characteristics of each MDS patient from our center were showed in the Supplementary Table 1. The baseline characteristics of the patients’ cohorts involved in our study were summarized in the Supplementary Table 2 and 3. The raw RNA-seq data (GSA-Human: HRA004641) and m^6^A-seq data (GSA-Human: HRA004643) had been deposited in the Genome Sequence Archive in National Genomics Data Center, China National Center for Bioinformation / Beijing Institute of Genomics, Chinese Academy of Sciences which are publicly accessible at https://ngdc.cncb.ac.cn/gsa-human. All other data are available from the corresponding author upon reasonable request.
