# Supplementary material for: The m6A methyltransferase METTL14 promotes cell proliferation via SETBP1-mediated activation of PI3K-AKT signaling pathway in myelodysplastic neoplasms
Source: Leukemia. 2024 Jul 25;38(10):2246–58. doi: 10.1038/s41375-024-02350-3 (PMC11436359; doi:10.1038/s41375-024-02350-3)
Supplement: Supplementary file 1 — Supplemental Methods [file 41375_2024_2350_MOESM1_ESM.docx]

**Supplemental Methods**

**Cytogenetic analysis**

Unstimulated bone marrow cells were obtained upon initial diagnosis. Cytogenetic slides were prepared using a standard protocol, and then R-banded. Metaphases were analyzed and the karyotypes were described according to the current International System for Human Cytogenetic Nomenclature [1]**.**

**Cell culture**

The MDS-L cell line, which is an internationally recognized MDS cell line developed from a MDS patient during the MDS disease phase [2, 3], was used in this research. The MDS-L cells were obtained as previously described [4], and confirmed through short tandem repeat (STR) analysis (Supplementary Figure 1 of the Supplemental Methods). MDS-L cells were cultured in RPMI 1640 medium supplemented with 10% fetal bovine serum (FBS) and 30 ng/mL human interleukin-3 (IL-3). Additionally, HEK293 cells were cultured in DMEM medium supplemented with 10% FBS. The HL-60 leukemia cell line was obtained from the Shanghai Institute of Cell Biology (Shanghai, China). The SKM-1 leukemia cell line was kindly endowed by Professor Gang Huang (University of Texas Health Science Center of San Antonio Texas, USA). The MOLM-13, MV4-11, Kasumi-1, AMLOCI-2, and U937 leukemia cell lines were kindly gifted by Professor Jie Jin (Zhejiang University, China). Leukemia cell lines were cultured in 1640 medium supplemented with 10% FBS. These cells were all cultured in a humidified incubator at 37 ℃ with 5% CO_2_. CD34^+^ cells extracted from the BM-MNCs of MDS patients were maintained in StemSpan^TM^ SFEM II medium supplemented with recombinant human SCF (50 ng/mL), Flt3 ligand (100 ng/mL), TPO (100 ng/mL), IL-3 (25 ng/mL), and IL-6 (10 ng/mL).

**RNA-seq and data analysis**

RNA-seq analysis was conducted by Cloudseq Biotech (China). Briefly, RNA libraries were prepared using the NEBNext^®^ Ultra^TM^ II Directional RNA Library Prep Kit (New England Biolabs, USA). The libraries were then sequenced on the Illumina Hiseq platform, generating 150 bp paired-end reads. Differentially expressed genes (DEGs) were identified based on a significance threshold of P < 0.05 and a fold-change in expression ≥ 2. The raw RNA-seq data had been deposited in the Genome Sequence Archive [5] in National Genomics Data Center [6], China National Center for Bioinformation / Beijing Institute of Genomics, Chinese Academy of Sciences (GSA-Human: HRA004641) which are publicly accessible at [https://ngdc.cncb.ac.cn/gsa-human](https://ngdc.cncb.ac.cn/gsa-human).

**m^6^A-seq and data analysis**

m^6^A-seq analysis was conducted by Cloudseq Biotech (China). Briefly, m^6^A RNA immunoprecipitation was performed using the GenSeq^TM^ m^6^A RNA IP Kit (GenSeq, China). Both the input samples and the m^6^A IP samples were prepared for library construction using the NEBNext^®^ Ultra^TM^ II Directional RNA Library Prep Kit (New England Biolabs, USA). The quality of the library was assessed using the BioAnalyzer 2100 system (Agilent Technologies). Library sequencing was carried out on an Illumina Hiseq instrument, generating 150 bp paired-end reads. Differentially methylated RNA sites were identified based on a significance threshold of P < 0.00001 and an absolute fold-change in methylation ≥ 2. The raw m^6^A-seq data had been deposited in the Genome Sequence Archive [5] in National Genomics Data Center [6], China National Center for Bioinformation / Beijing Institute of Genomics, Chinese Academy of Sciences (GSA-Human: HRA004643) which are publicly accessible at [https://ngdc.cncb.ac.cn/gsa-human](https://ngdc.cncb.ac.cn/gsa-human).

**Lentivirus production and infection**

The human METTL14 lentiviral small hairpin RNAs (shRNAs) were subcloned into the psi-LVRU6GP vector (GeneCopoeia, USA). A Dox-inducible shMETTL14 (shMETTL14_Tet-on) based on the pTSB-Tight-shRNA (miR30 loop)-EF1-TetR-2A-PURO was designed and constructed by Transheep (Shanghai, China). The lentiviral shRNAs targeting METTL3 and SETBP1 based on the pTSH-SH-copGFP-2A-PURO vector, the overexpression plasmids of pCDH-3×Flag-METTL14, pCDH-3×Flag-METTL14-R298P, pCDH-3×Flag-SETBP1 were also designed and constructed by Transheep (Shanghai, China). HER-293T cells were transfected with the aforementioned plasmids and virus packing plasmids using the DNA transfection reagent (Neofect, China). After a 2-day incubation period, the virus was collected and subsequently introduced onto the MDS-L cells for a duration of 3 days. Following this, the infected cells were subjected to selection with 2 μg/mL puromycin for a duration of at least 2 days. Western blot, m^6^A dot blot assay, m^5^C dot blot assay were conducted on day 6 post-transduction, while assessments of cell cycle and apoptosis were carried out on day 6 and 10 post-transduction, respectively. Cell proliferation assay and clonal formation assay were performed on day 8 post-transduction. Cells transduced with the Dox-inducible shMETTL14 (shMETTL14_Tet-on) need to culture with or without Dox (2 μg/mL) for another 48h after puromycin selection. The specific target sequences for the shRNAs are shown in Supplementary Table 1 of the Supplemental Methods.

**m^6^A colorimetric quantification**

The RNA was extracted using Trizol (TakaRa, Japan) and quantified using NanoDrop 2000. The RNA m^6^A level was assessed using the m^6^A RNA methylation quantification kit (P-9005, EpiGentek, USA) according to the manufacturer's instructions.

**m^6^A and** **m^5^C dot blot assay**

The m^6^A and m^5^C dot blot assay was performed following a previously published protocol [7]. Briefly, 100 - 400 ng of RNA was applied onto Amersham Hybond-N^+^ membranes (GE Healthcare, USA) using the Bio-Dot Apparatus (Bio-Rad, USA) under vacuum. The membranes were subsequently crosslinked using UV irradiation and stained with a solution containing 0.3M sodium acetate and 0.02% methylene blue. Following blocking with 5% nonfat milk, the membranes were incubated overnight at 4℃ with a 1:1000 dilution of m^6^A antibody (202003, Synaptic Systems, Germany) or m^5^C antibody (ab214727, Abcam, USA). The membranes were then washed three times with 1×PBST and incubated with a secondary antibody (1:5000 dilution; CST, USA). Finally, the membranes were visualized using an ECL kit (Thermo Fisher Scientific, USA) and detected using the ChemiDoc MP Imaging System (Bio-Rad, USA).

**Western blot**

The Western blot procedure was conducted according to previously established methods [4]. Antibodies used for Western blot were as follows: METTL4 (51104S, CST, USA), SETBP1 (PA5-77171, Invitrogen, USA), METTL3 (15073-1-AP, Proteintech, China), FLAG (F1804, Sigma-Aldrich, USA), PI3K (ab32089, Abcam, USA), p-PI3K^Y464^ (ab138364, Abcam, USA), AKT (60203-2-Ig, Proteintech, China), p-AKT^Y473^ (66444-1-Ig, Proteintech, China), GAPDH (60004-1-Ig, Proteintech, China), and HRP-conjugated secondary antibodies (CST, USA). The primary antibodies were diluted at a ratio of 1:1000, while the secondary antibodies were diluted at a ratio of 1:5000.

**q-PCR**

RNA extraction was performed using Trizol (TakaRa, Japan), and cDNA synthesis was carried out using the PrimerScript RT agent Kit (TakaRa, Japan) following the manufacturer's instructions. The amplified cDNA was quantitatively assessed using the Applied Biosystems CFX96 real-time PCR system (Bio-Rad, USA) and the TB-Green PCR Master Mix kit (TakaRa, Japan). The relative gene expression was determined using the 2^−ΔΔCt^ method and normalized to the expression of GAPDH. The primers were listed in Supplementary Table 2 of the Supplemental Methods.

**Cell proliferation assay**

The cells were seeded in triplicate at a density of 2000 cells/100 μL in 96-well plates at 37 ℃ in a humidified incubator with 5% CO_2_. Cell proliferation ability was evaluated after a 2, 4, and 6-day incubation period using the CellTiter-Lumi^TM^ Assay (Beyotime biotechnology, China) in accordance with the manufacturer's instructions. Briefly, 100 μL of CellTiter-Lumi^TM^ reagents were added. After shaking for 2 minutes and incubating at room temperature for 10 minutes, the luminescence was examined by a microplate reader (Thermo Fisher Scientific, USA).

**Colony formation assay**

The colony formation assay was conducted following a published protocol with slight modifications [8]. Five hundred cells were seeded in 3.5 cm dished with 1 mL of medium consisting of 400 μL of methylcellulose basal medium (STEMCELL Technologies, Canada), 200 μL FBS, 50 ng/mL IL-3, and 2 μg/mL puromycin. The colonies were quantified after a 14-day incubation period at 37 ℃ in a humidified incubator with 5% CO_2_.

**Flow cytometry**

Cell apoptosis was evaluated using an apoptosis detection kit (Multiscience, China) according to the manufacturer's instructions. Briefly, the cells were stained with Annexin V and PI for 5 minutes in the absence of light. Cell cycle was assessed using a cell cycle staining kit (Multiscience, China) in accordance with the manufacturer's guidelines. Specifically, cells were stained with PI for 30 minutes at room temperature in the dark. Fluorescence intensity of PI, indicative of DNA content, was utilized to identify the different phases of the cell cycle. Flow cytometric analyses of cell apoptosis and cell cycle were performed on FACScan^TM^ flow cytometer (Becton Dickinson, USA), and data were analyzed by NovoExpress software (ACEA Biosciences, USA). Brilliant Violet 421^TM^ anti-human CD45 antibody (Biolegend, USA) was used for detecting the proportions of human CD45^+^ cells. Flow cytometric analysis of the proportions of human CD45^+^ cells were performed on Fortessa X-20 (Becton Dickinson, USA), and data were analyzed by FlowJo. The gating strategy for cell apoptosis, cell cycle, and the percentages of hCD45^+^ in our study were as shown in the Supplementary Figure 2 - 4 of the Supplemental Methods.

**Methylated RNA immunoprecipitation (MeRIP)-qPCR**

MeRIP-qPCR was performed using the GenSeq^TM^ m^6^A RNA IP Kit (GenSeq, China) following the manufacturer’s instruction. The fragmented total RNA was subjected to incubation with PGM Beads conjugated with either IgG or m^6^A antibody for a duration of 1 hour at a temperature of 4℃. A portion of the fragmented RNA was retained as input. Following washing with IP buffer, the RNA was eluted from the beads and extracted. Subsequently, the m^6^A-enriched RNA, IgG-enriched RNA, and the input RNA were subjected to reverse transcription into cDNA and subsequently analyzed using q-PCR. The enrichment of immunoprecipitated RNA in each sample was determined by normalizing it to the input sample. The primers for this experiment were listed in Supplementary Table 3 of the Supplemental Methods.

**RNA immunoprecipitation (RIP)-qPCR**

RIP-qPCR was performed using the Magna RIP RNA-Binding Protein Immunoprecipitation Kit (Sigma-Aldrich, USA) following the manufacturer’s instruction. Initially, magnetic beads were coated with 5 μg METTL14 antibody (HPA038002, Sigma-Aldrich, USA) or rabbit IgG antibody, followed by overnight incubation with cell lysates at 4℃. The immunoprecipitated complexes were washed, subjected to proteinase K digestion and RNA purification. The METTL14-enriched RNA, IgG-enriched RNA and the input RNA were then reverse transcribed into cDNA and analyzed using q-PCR. The enrichment of immunoprecipitated RNA was normalized to the input sample. The primers were listed in Supplementary Table 4 of the Supplemental Methods.

**Dual-Luciferase reporter assay**

The dual-luciferase reporters including SETBP1-3′UTR-wild type (WT) and SETBP1-3′UTR-mutant type (MT) were constructed by Transheep (Shanghai, China). The SETBP1 WT 3′-UTR sequences (NM_015559; 8762-9216) were amplified and cloned into the psiCHECK2 vector to construct SETBP1-3′UTR-WT reporter. For mutant reporter plasmid, adenosine (A) in m^6^A motifs (at positions 8800, 9046, and 9168) was replaced by thymine (T). Subsequently, HEK293 cells were seeded in 24-well plates and transfected with plasmids of METTL14, SETBP1-3′UTR-WT reporter or SETBP1-3′UTR-MT reporter using DNA transfection reagent. The activities of firefly luciferase and Renilla luciferase in each well were measured using a Dual-luciferase Reporter Assay Kit (Promega, Madison, WA, USA) after a 48-hour incubation period.

**RNA stability assay**

The RNA stability assay was conducted following a previously published protocol with some modifications [8]. MDS-L cells were treated with mRNA transcription inhibitor actinomycin D (Adooq Bioscience, USA) at a concentration of 5 μg/mL and collected at preset time points. RNA was extracted using Trizol (TakaRa, Japan) and analyzed using q-PCR. The expression of GAPDH was used as endogenous control. The remaining relative mRNA levels of SETBP1 at 1.5 h and 3 h were normalized to 0 h, and the mRNA's half-life was then calculated. The linear fitting curves based on simple linear regression were generated for each group using GraphPad Prism 9.0.

**Co-immunoprecipitation assay**

Cells were collected and lysed with 800 μL NP40 lysis in 1.5 mL Eppendorf tube. Then lysate was divided into two groups equally. 5 μg FLAG antibody (F1804, Sigma-Aldrich, USA) and 5 μg normal mouse IgG (sc-2025, SantaCruz, USA) were added into the two groups respectively. Each group was incubated in 4℃ for 12 hours, and then incubated with 20 μL protein G magnetic beads in 4℃ for 16 hours. After incubation, the supernatants were removed and the beads were washed with 1 mL of NP40 lysis buffer for 4 times. The Co-IP proteins were eluted with loading buffer and followed by Western blot analysis.

**Bioinformatic analyses of public databases**

The public MDS cohort was firstly used to identify the differentially expressed m^6^A regulators among groups categorized by the percentages of bone marrow blasts (healthy donors vs blasts < 5% vs blasts ≥ 5%) using the Kruskal-Wallis’s test. The expressions of m^6^A regulators were demonstrated by a heatmap using R language. The optimal cut-off values of gene expressions of m^6^A regulators for survival analyses were determined by R2 Genomics Analysis and Visualization Platform (http://r2.amc.nl). Then the univariate Cox analysis was used to evaluate the associations between expressions of m^6^A regulators and OS, followed by a multivariate Cox regression with a stepwise approach if p < 0.1 in the univariate analysis. Other variables included in the multivariate Cox model were age, gender, and the International Prognostic Scoring System (IPSS). Gene mutation status with p < 0.1 in the univariate analysis was also included in the multivariate analysis. Then the Venn diagram was used to find the key m^6^A regulator whose expression level was not only related with the percentages of bone marrow blasts but also independently associated with prognosis.

**References of the supplemental methods**

1. Shaffer LG SM, Campbell LJ. ISCN 2013: An International System for Human Cytogenetic Nomenclature. 2013.

2. Nakamura S, Ohnishi K, Yoshida H, Shinjo K, Takeshita A, Tohyama K, et al. Retrovirus-mediated gene transfer of granulocyte colony-stimulating factor receptor (G-CSFR) cDNA into MDS cells and induction of their differentiation by G-CSF. Cytokines Cell Mol Ther. 2000;6(2):61-70.

3. Drexler HG, Dirks WG, Macleod RA. Many are called MDS cell lines: one is chosen. Leuk Res. 2009;33(8):1011-6.

4. Wang L, Zhang Q, Ye L, Ye X, Yang W, Zhang H, et al. All-trans retinoic acid enhances the cytotoxic effect of decitabine on myelodysplastic syndromes and acute myeloid leukaemia by activating the RARalpha-Nrf2 complex. Br J Cancer. 2023;128(4):691-701.

5. Chen T, Chen X, Zhang S, Zhu J, Tang B, Wang A, et al. The Genome Sequence Archive Family: Toward Explosive Data Growth and Diverse Data Types. Genomics Proteomics Bioinformatics. 2021;19(4):578-83.

6. Members C-N, Partners. Database Resources of the National Genomics Data Center, China National Center for Bioinformation in 2023. Nucleic Acids Res. 2023;51(D1):D18-D28.

7. Li Z, Weng H, Su R, Weng X, Zuo Z, Li C, et al. FTO Plays an Oncogenic Role in Acute Myeloid Leukemia as a N(6)-Methyladenosine RNA Demethylase. Cancer Cell. 2017;31(1):127-41.

8. Weng HY, Huang HL, Wu HZ, Qin X, Zhao BXS, Dong L, et al. METTL14 Inhibits Hematopoietic Stem/Progenitor Differentiation and Promotes Leukemogenesis via mRNA m(6)A Modification. Cell Stem Cell. 2018;22(2):191-+.

**Supplementary Tables of the Supplemental Methods**

**Supplementary Table 1 of the Supplemental Methods. The target sequences for shRNAs**

| **shRNA** | **Target Sequence (5’ - 3’)** |
| --- | --- |
| Scramble-METTL14 | GCTTCGCGCCGTAGTCTTA |
| shMETTL14-#1 | GCCGTGTTAAATAGCAAAGAT |
| shMETTL14-#2 | GCATTGGTGCCGTGTTAAATA |
| Scramble-SETBP1, METTL3 | GATTCTCCGAACGTGTCACGT |
| shSETBP1-#1 | TCCGGTGCAGCTAAGCATAAA |
| shSETBP1-#2 | CGTGTCCCTAAGTTGAGTAAA |
| shMETTL3-#1 | GACCGAATGAAGGTCCACATT |
| shMETTL3-#2 | GCAGTCCATTGATAAGAGTTA |

**Supplementary Table 2 of the Supplemental Methods. The primer sequences for q-PCR**

| **Gene** |  | **Sequence (5’ - 3’)** |
| --- | --- | --- |
| **GAPDH** | Forward | AATCCCATCACCATCTTCCAG |
|  | Reverse | AAATGAGCCCCAGCCTTC |
| **METTL14** | Forward | GAGTGTGTTTACGAAAATGGGGT |
|  | Reverse | CCGTCTGTGCTACGCTTCA |
| **SETBP1** | Forward | GCCAGCCGCAGTTGACAGTG |
|  | Reverse | CCGCCGCTTGAACCTCTTCTTC |

**Supplementary Table 3 of the Supplemental Methods. The primer sequences of SETBP1 for MeRIP-qPCR**

| **Gene** |  | **The primer sequences for MeRIP-qPCR (5’ - 3’)** |
| --- | --- | --- |
| **SETBP1** | Forward | AACCTTTGCTCTCCACTCCA |
|  | Reverse | TCCCTCTGTGAAGTTTGCCT |

**Supplementary Table 4** **of the Supplemental Methods. The primer sequences of SETBP1 for RIP-qPCR**

| **Gene** |  | **The primer sequences for RIP-qPCR (5’ - 3’)** |
| --- | --- | --- |
| **SETBP1** | Forward | GCCAGCCGCAGTTGACAGTG |
|  | Reverse | CCGCCGCTTGAACCTCTTCTTC |

**Supplementary Figures of the Supplemental Methods**

**Supplementary Figure 1 of the Supplemental Methods**

**
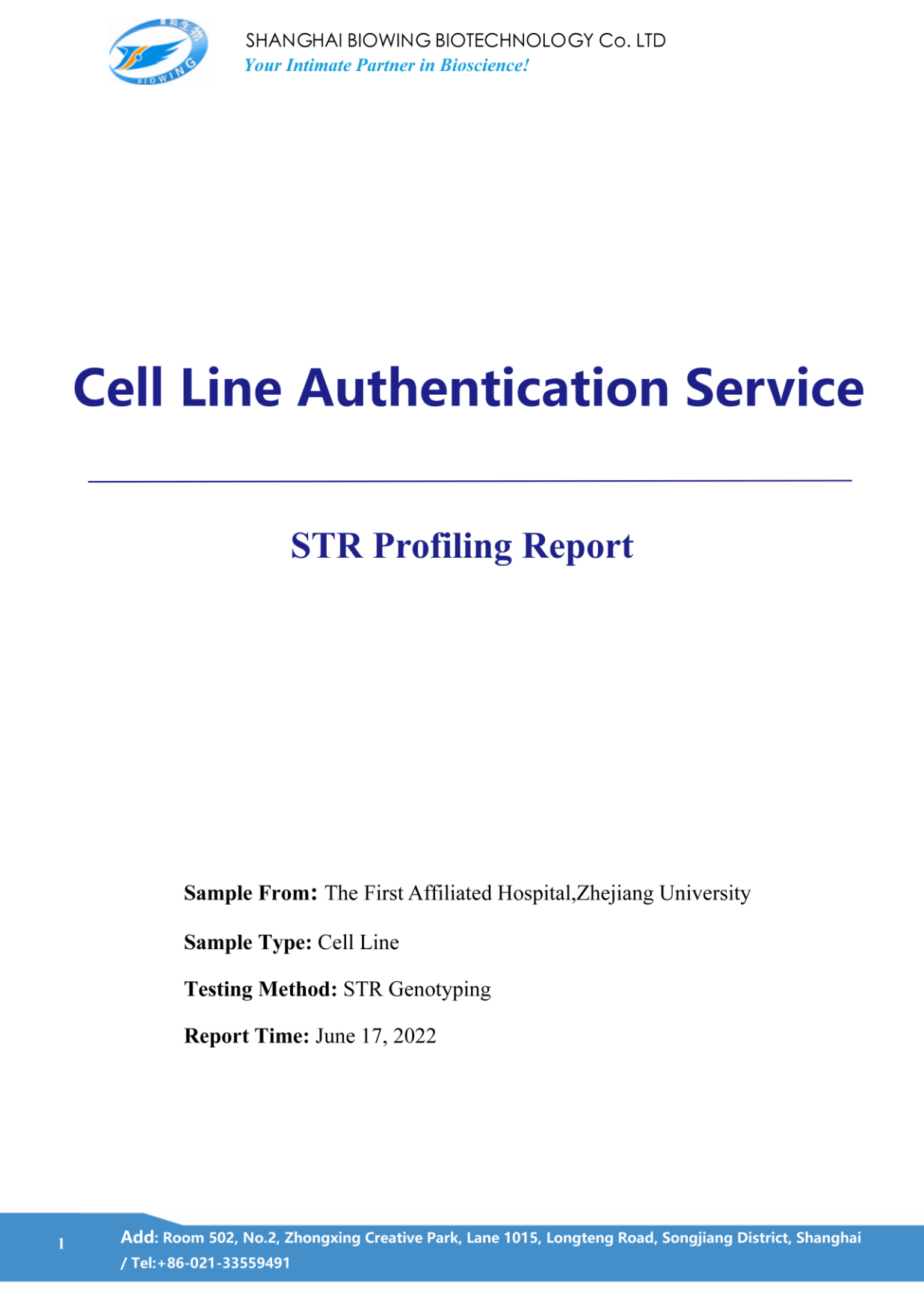
**

**
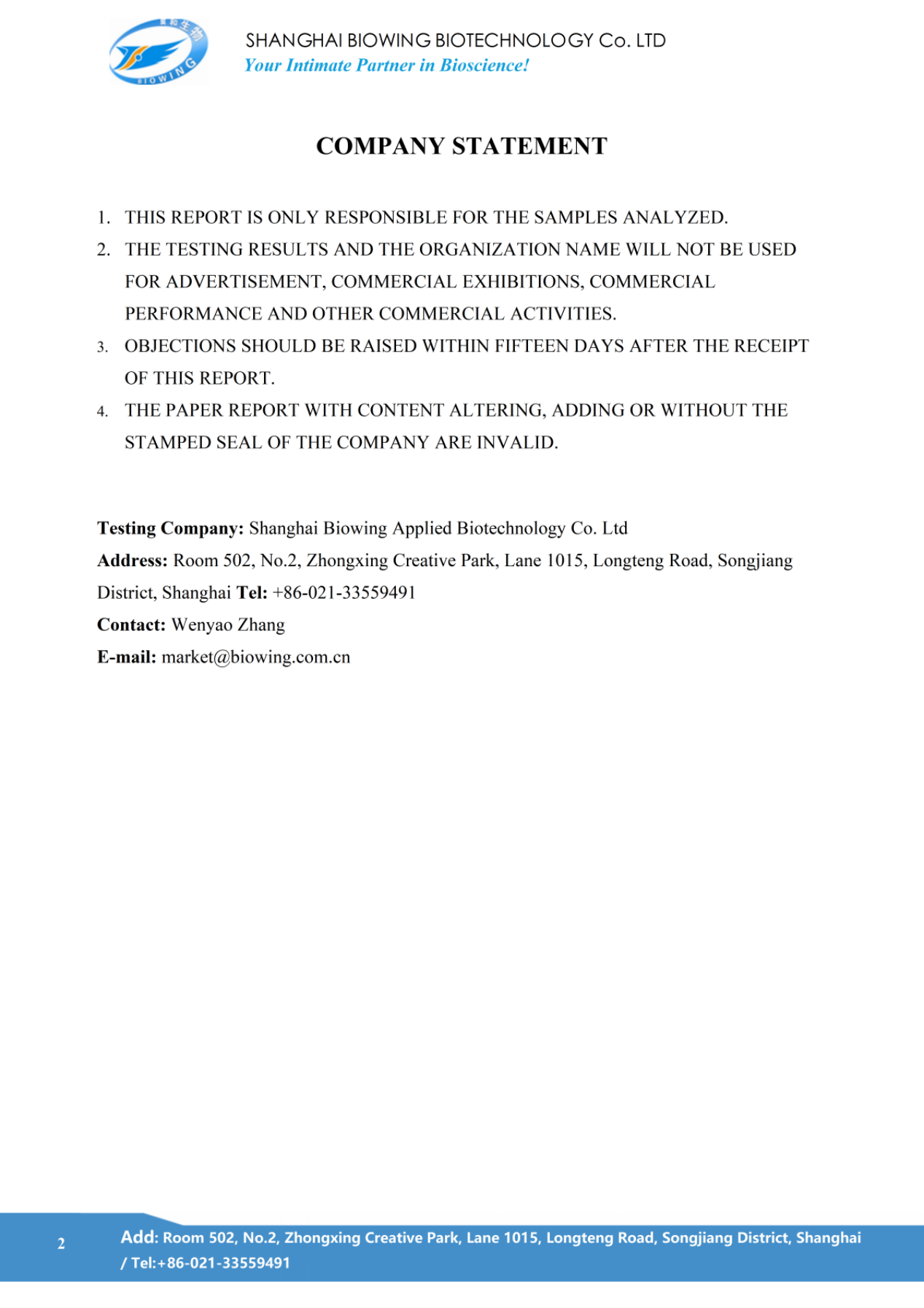
**

**
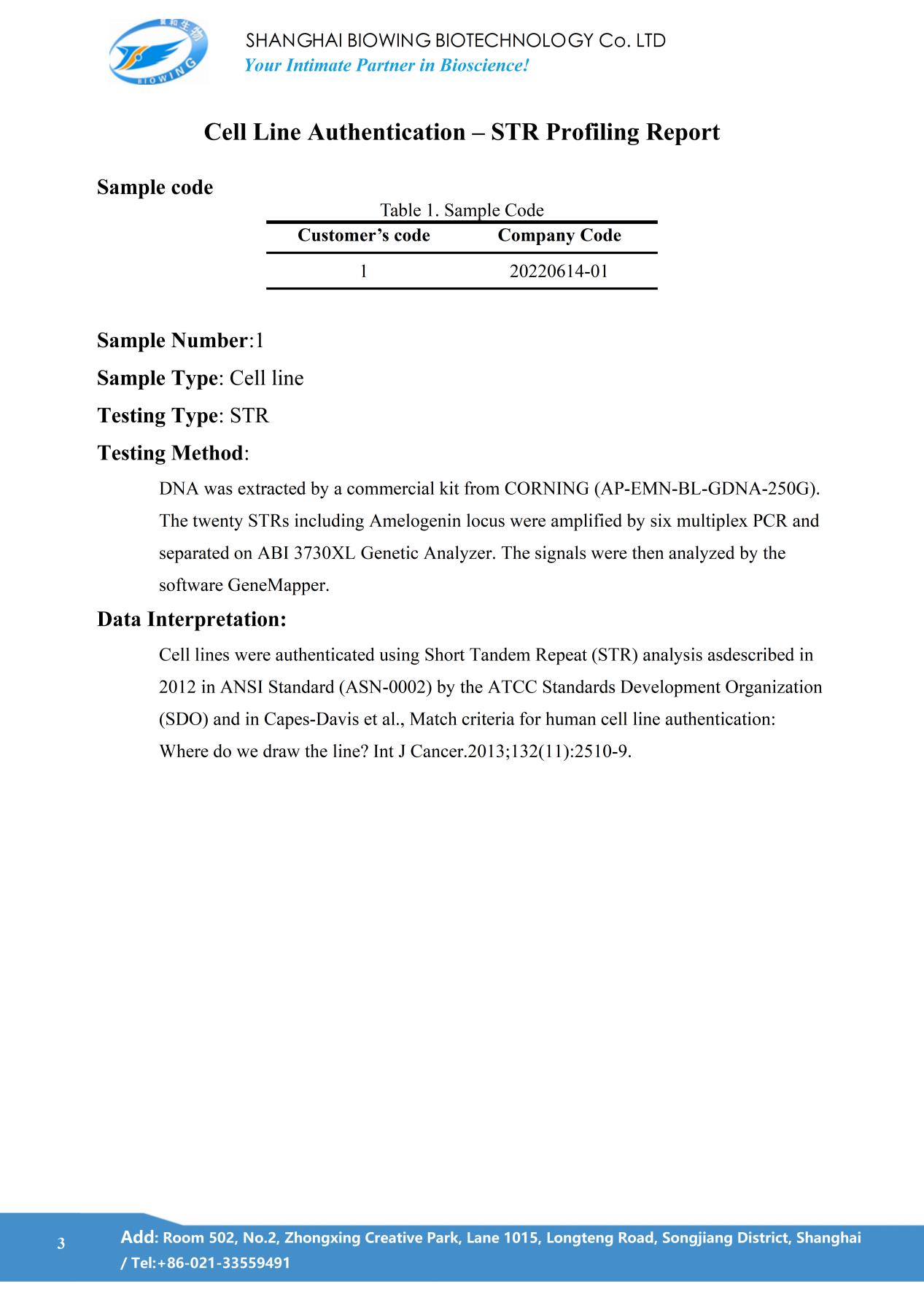
**

**
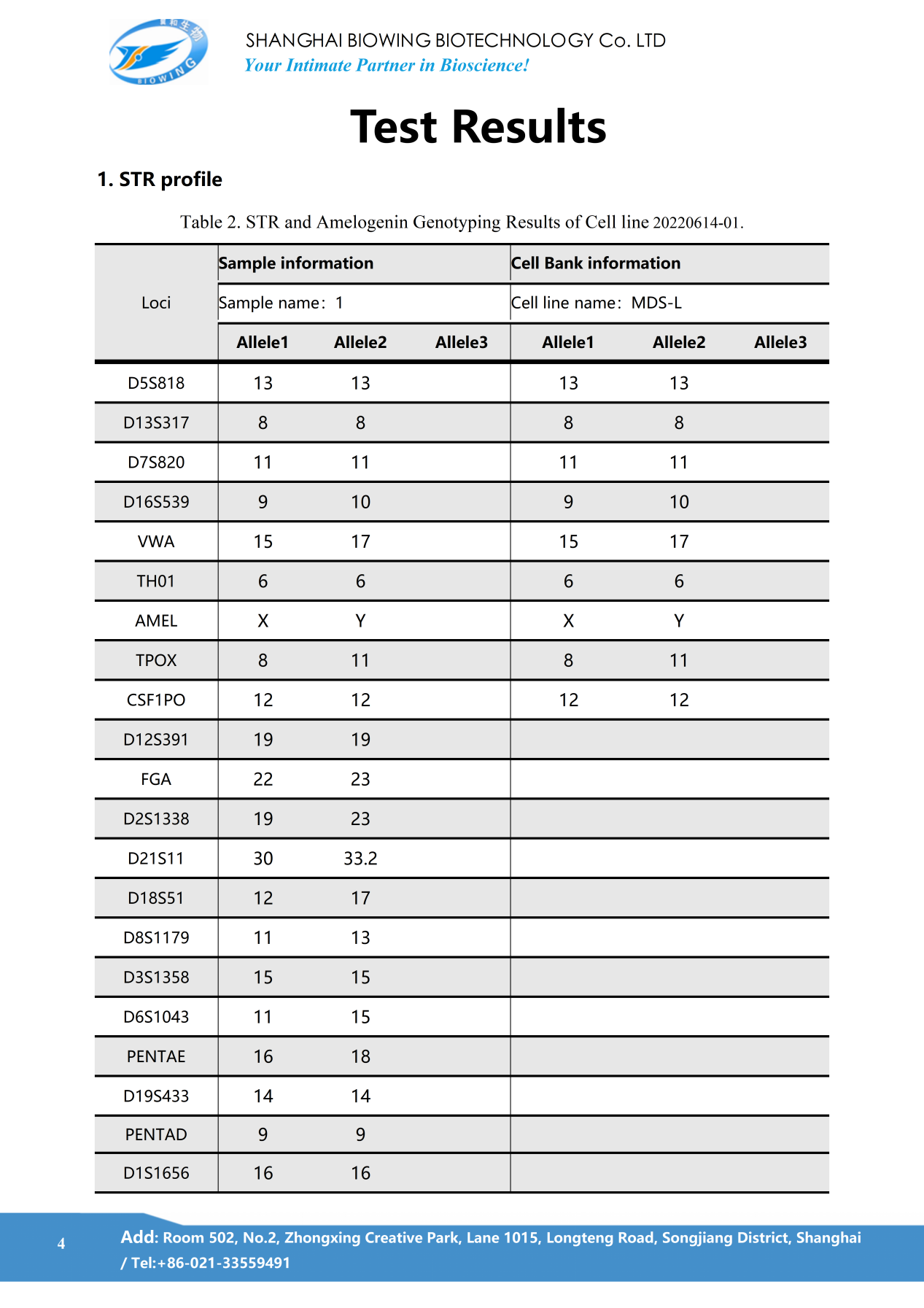
**

**
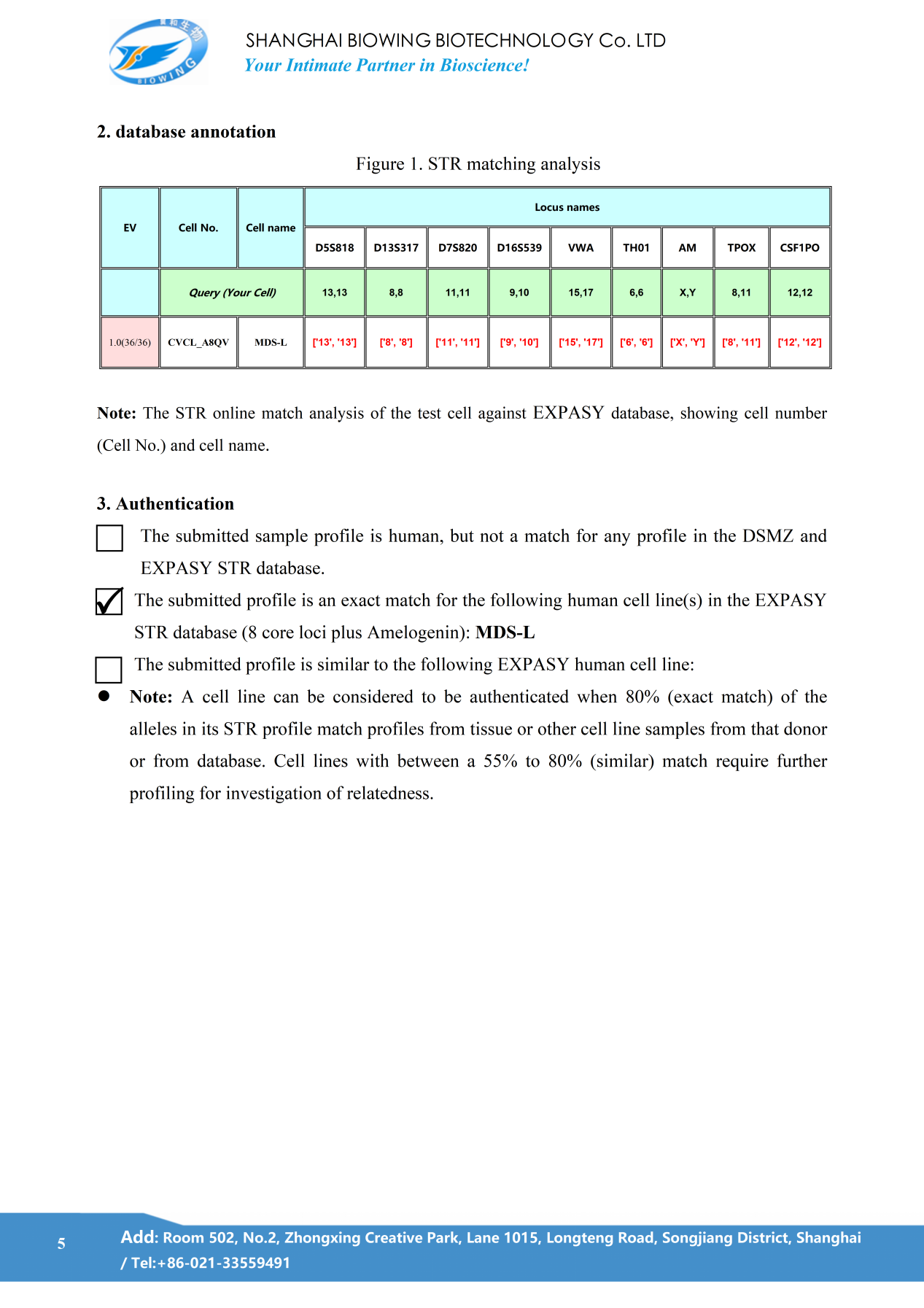
**

**
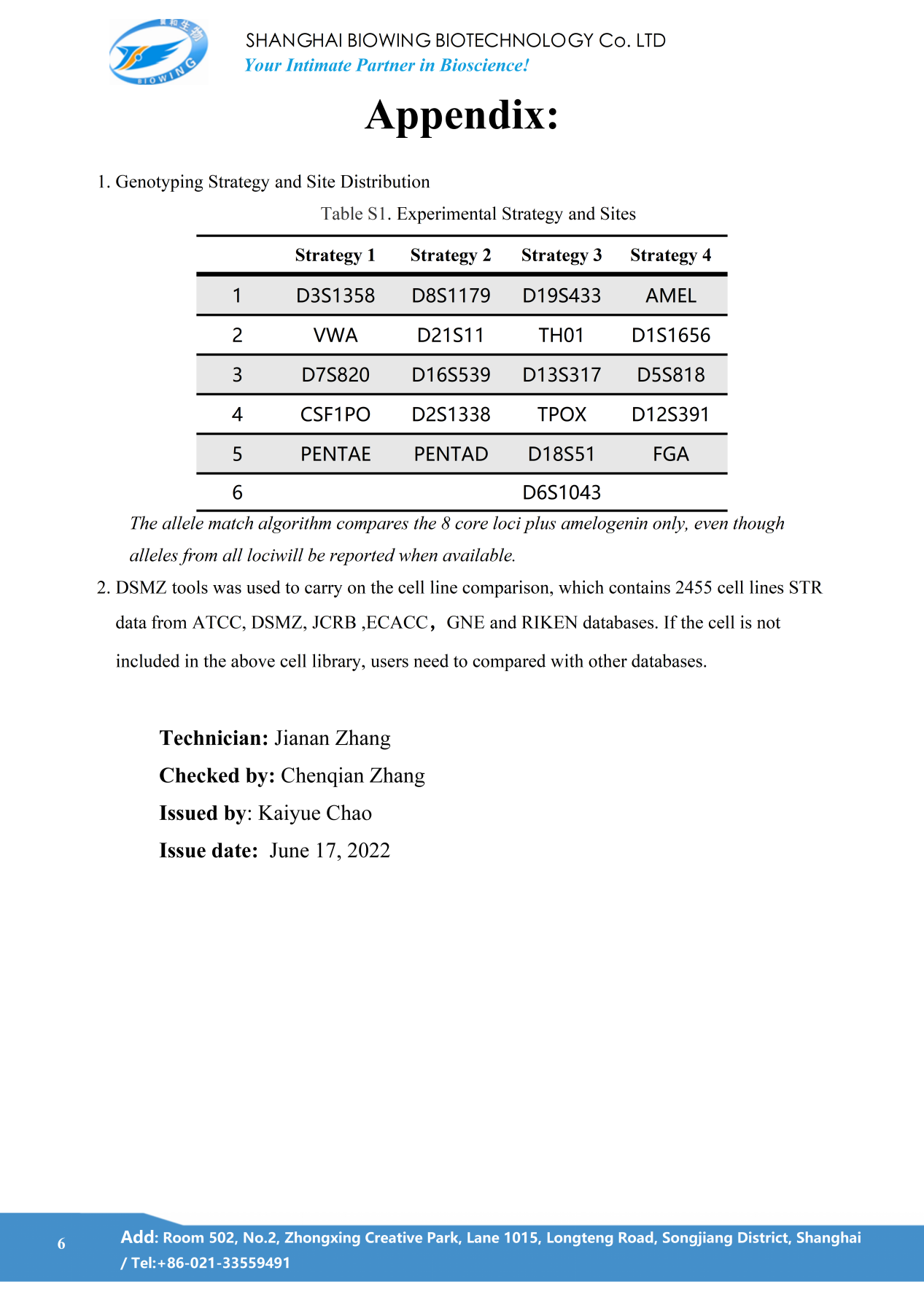
**

**Supplementary Figure 1. MDS ce­­ll line MDS-L validated by short tandem repeat (STR) profiling.**

**Supplementary Figure 2 of the Supplemental Methods**


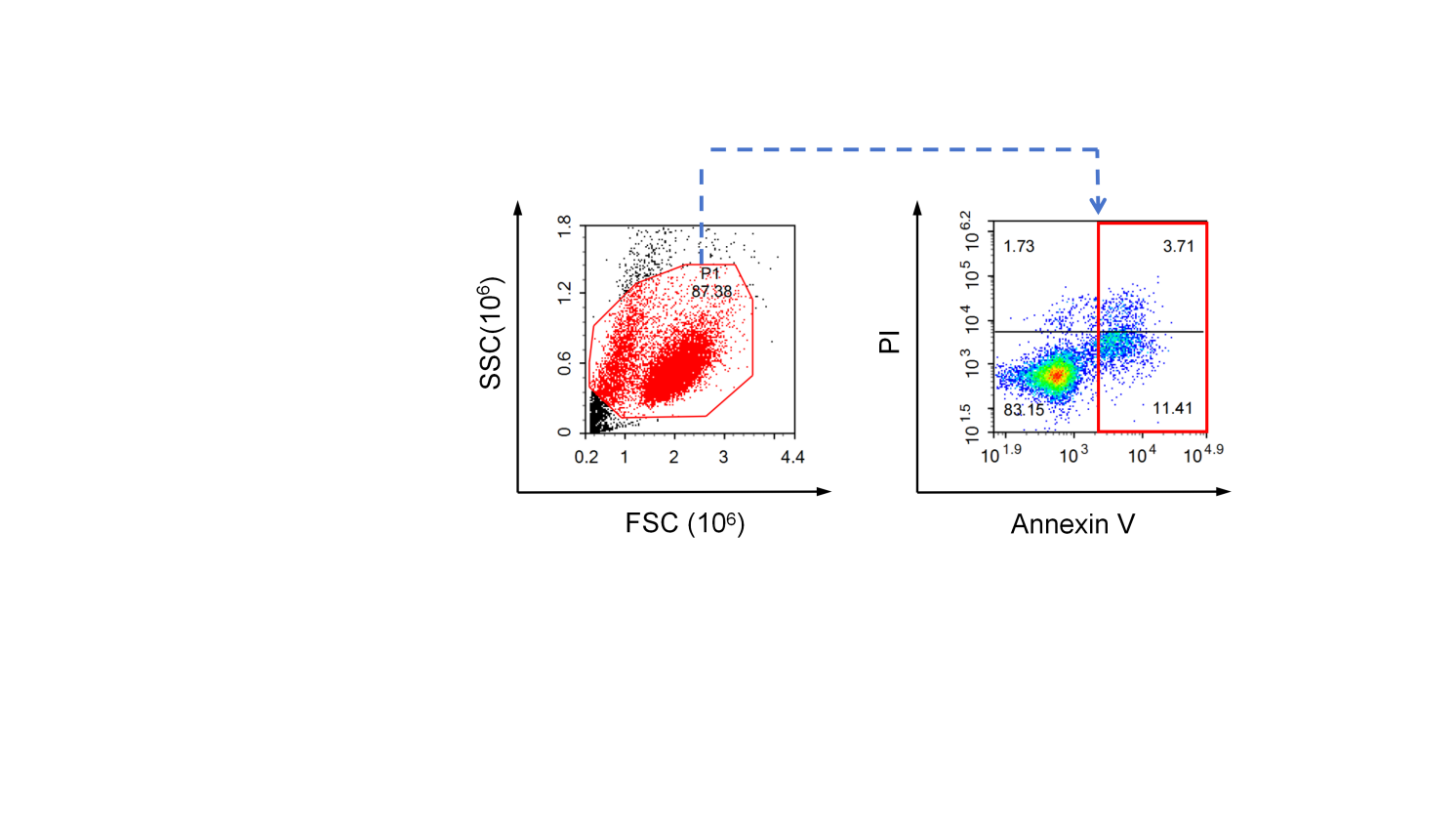


**Supplementary Figure 2. The gating strategy for** **cell apoptosis.**

**Supplementary Figure 3 of the Supplemental Methods**


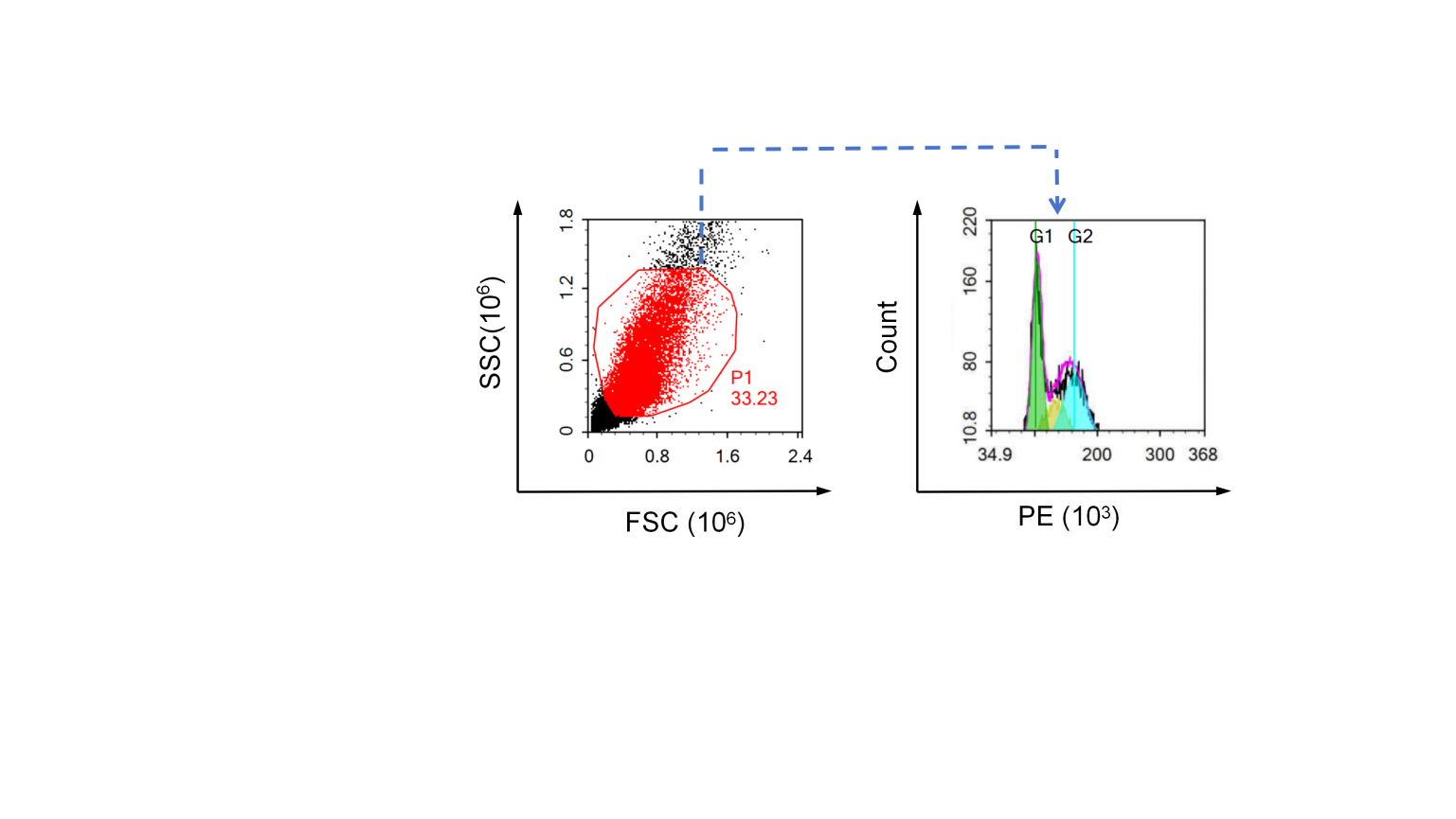


**Supplementary Figure 3. The gating strategy for cell cycle.**

**Supplementary Figure 4 of the Supplemental Methods**


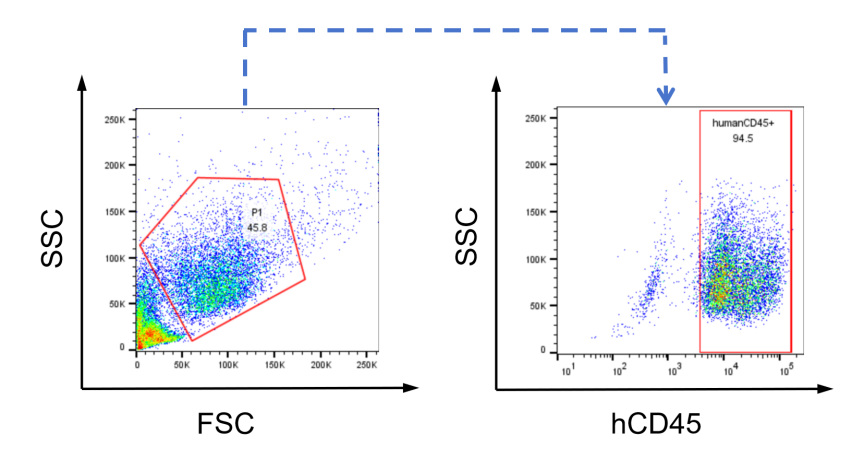


**Supplementary Figure 4. The gating strategy for the percentages of hCD45^+^ cells.**
