# Supplementary material for: The m6A methyltransferase METTL14 promotes cell proliferation via SETBP1-mediated activation of PI3K-AKT signaling pathway in myelodysplastic neoplasms
Source: Leukemia. 2024 Jul 25;38(10):2246–58. doi: 10.1038/s41375-024-02350-3 (PMC11436359; doi:10.1038/s41375-024-02350-3)
Supplement: Supplementary file 3 — Supplementary Table 2-6 [file 41375_2024_2350_MOESM3_ESM.docx]

**Supplementary Table 2-6**

**Supplementary Table 2. Demographic and baseline characteristics of the MDS cohorts from our center**

| Variable | Cohort for detection of global m^6^A level in BM-MNCs  (N=29) | Cohort for examination of METTL14 level in CD34^+^ cells (N=16) | Cohort for examination of METTL14 level in BM-MNCs (N=221) | Cohort for examination of SETBP1 level in BM-MNCs (N=152) | P |
| --- | --- | --- | --- | --- | --- |
| Age (yrs) |  |  |  |  | 0.343^#^ |
| Median (range) | 59 (21-78) | 66 (25-82) | 59 (15-88) | 57 (17-88) |  |
| Gender, N (%) |  |  |  |  | 0.666^$^ |
| Male | 16 (55.2) | 10 (62.5) | 143 (64.7) | 102 (67.1) |  |
| Female | 13 (44.8) | 6 (37.5) | 78 (35.3) | 50 (32.9) |  |
| Bone marrow blasts |  |  |  |  | 0.366^$^ |
| < 5%, N (%) | 18 (62.1) | 6 (37.5) | 129 (58.4) | 91 (59.9) |  |
| ≥ 5%, N (%) | 11 (37.9) | 10 (62.5) | 92 (41.6) | 61 (40.1) |  |
| Karyotype |  |  |  |  | 0.312^$^ |
| Normal, N (%) | 13 (44.8) | 8 (50.0) | 133 (60.2) | 94 (61.8) |  |
| Abnormal, N (%) | 16 (55.2) | 8 (50.0) | 88 (39.8) | 58 (38.2) |  |
| IPSS-R |  |  |  |  | 0.874^$^ |
| ≤ 3.5, N (%) | 10 (34.5) | 6 (37.5) | 92 (41.6) | 64 (42.1) |  |
| > 3.5, N (%) | 19 (65.5) | 10 (62.5) | 129 (58.4) | 88 (57.9) |  |
| Follow-up treatment |  |  |  |  | 0.637^$^ |
| Supportive care, N (%) | 19 (65.5) | 6 (37.5) | 121 (54.8) | 90 (59.2) |  |
| HMAs monotherapy, N (%) | 5 (17.2) | 7 (43.8) | 55 (24.9) | 30 (19.7) |  |
| HMAs + other agents, N (%) | 4 (13.8) | 3 (18.8) | 37 (16.7) | 26 (17.1) |  |
| Chemotherapy, N (%) | 1 (3.4) | 0 (0.0) | 8 (3.6) | 6 (3.9) |  |

BM-MNCs, Bone marrow mononuclear cells; HMAs, hypomethylating agents; IPSS-R, revised International Prognostic Scoring System; ^#^P value calculated using the Kruskal-Wallis’s test; ^$^P value calculated using the Pearson’s chi-squared test.

**Supplementary Table 3. Demographic and baseline characteristics of the** **publicly available MDS cohort (GSE58831)**

| Variable | Baseline distribution in cohort (N=113) |
| --- | --- |
| Age (yrs) |  |
| Median (range) | 67 (19-87) |
| Gender, N (%) |  |
| Male | 77 (68.1) |
| Female | 36 (31.9) |
| Bone marrow blasts |  |
| < 5%, N (%) | 63 (55.8) |
| ≥ 5%, N (%) | 50 (44.2) |
| Karyotype |  |
| Normal, N (%) | 51 (51/107, 47.7) |
| Abnormal, N (%) | 56 (56/107, 52.3) |
| IPSS, N (%) |  |
| low | 30 (30/106, 28.3) |
| Int-1 | 48 (48/106, 45.3) |
| Int-2 | 22 (22/106, 20.8) |
| High | 6 (6/106, 5.7) |

Int-1, intermediate 1; Int-2, intermediate 2; IPSS, International Prognostic Scoring System

**Supplementary Table 4. Multivariate Cox regression analysis for overall survival (OS) of MDS cohort with examination of METTL14 level in BM-MNCs**

| **Variables** | **P** | **HR^1^** | **95% CI** |
| --- | --- | --- | --- |
| IPSS-R (HR^2^ vs LR) | 0.003 | 2.241 | 1.316 - 3.818 |
| Age ( ≥ vs < 60 years) | 0.008 | 2.099 | 1.212 - 3.635 |
| Gender (male vs female) | 0.987 | 0.995 | 0.519 - 1.908 |
| METTL14 (high vs low) | 0.025 | 1.932 | 1.086 - 3.440 |
| *SF3B1* (MT vs WT) | 0.482 | 0.701 | 0.260 - 1.888 |
| *FLT3* (MT vs WT) | 0.164 | 3.008 | 0.639 - 14.158 |
| *CEBPA* (MT vs WT) | 0.611 | 0.723 | 0.208 - 2.518 |
| *NRAS* (MT vs WT) | 0.627 | 1.358 | 0.396 - 4.661 |
| *ETV6* (MT vs WT) | 0.238 | 1.806 | 0.677 - 4.816 |
| *IDH2* (MT vs WT) | 0.822 | 1.154 | 0.331 - 4.025 |
| *TP53* (MT vs WT) | 0.016 | 2.511 | 1.191 - 5.293 |
| Follow-up treatment |  |  |  |
| HMAs monotherapy vs Supportive care | 0.280 | 0.323 | 0.041 - 2.513 |
| HMAs + other agents vs Supportive care | 0.712 | 0.683 | 0.090 - 5.179 |
| Chemotherapy vs Supportive care | 0.533 | 0.516 | 0.065 - 4.122 |

CI, confidence interval; HMAs, hypomethylating agents; HR^1^, hazards ratio; HR^2^, higher-risk; LR, lower-risk; MT, mutant type; WT, wild type.

**Supplementary Table 5. Multivariate Cox regression analysis for leukemic-free survival (LFS) of MDS cohort with examination of METTL14 level in BM-MNCs**

| **Variables** | **P** | **HR^1^** | **95% CI** |
| --- | --- | --- | --- |
| IPSS-R (HR^2^ vs LR) | 0.048 | 1.774 | 1.005 - 3.128 |
| Age ( ≥ vs < 60 years) | 0.030 | 1.854 | 1.060 - 3.240 |
| Gender (male vs female) | 0.829 | 1.071 | 0.572 - 2.005 |
| METTL14 (high vs low) | 0.006 | 2.320 | 1.271 - 4.233 |
| *CEBPA* (MT vs WT) | 0.780 | 1.169 | 0.390 - 3.503 |
| *DNMT3A* (MT vs WT) | 0.686 | 0.863 | 0.423 - 1.763 |
| *ETV6* (MT vs WT) | 0.104 | 2.245 | 0.848 - 5.946 |
| *IDH2* (MT vs WT) | 0.374 | 1.657 | 0.544 - 5.043 |
| *IKZF1* (MT vs WT) | < 0.001 | 33.618 | 6.242 - 181.073 |
| *KRAS* (MT vs WT) | 0.075 | 2.491 | 0.911 - 6.814 |
| *NPM1* (MT vs WT) | 0.022 | 4.403 | 1.236 - 15.684 |
| *NRAS* (MT vs WT) | 0.518 | 1.502 | 0.437 - 5.159 |
| *TP53* (MT vs WT) | 0.010 | 2.756 | 1.277 - 5.951 |
| Follow-up treatment |  |  |  |
| HMAs monotherapy vs Supportive care | 0.015 | 0.155 | 0.034 - 0.697 |
| HMAs + other agents vs Supportive care | 0.089 | 0.275 | 0.062 - 1.219 |
| Chemotherapy vs Supportive care | 0.065 | 0.231 | 0.049 - 1.098 |

CI, confidence interval; HMAs, hypomethylating agents; HR^1^, hazards ratio; HR^2^, higher-risk; IPSS-R, revised International Prognostic Scoring System; LR, lower-risk; MT, mutant type; WT, wild type.

**Supplementary Table 6. The correlations of METTL14 with potential key targets based on the publicly available MDS database (GSE58831)**

| **Potential targets** | **Correlation with METTL14 indicated by RNA-seq** | **Pearson’s correlation with METTL14 indicated by GSE58831 cohort** | |
| --- | --- | --- | --- |
|  |  | **r** | **P** |
| STARD8 | Positive | -0.186 | 0.049 |
| ZNF157 | Positive | 0.214 | 0.023 |
| NEURL1B | Positive | -0.053 | 0.575 |
| OTOF | Positive | -0.152 | 0.107 |
| CACNB1 | Positive | -0.558 | <0.0001 |
| CD93 | Positive | -0.020 | 0.832 |
| CD79B | Positive | -0.131 | 0.168 |
| LBH | Positive | -0.084 | 0.379 |
| MMRN1 | Positive | 0.127 | 0.181 |
| CGN | Positive | -0.241 | 0.010 |
| TIAM1 | Positive | -0.198 | 0.036 |
| SV2A | Positive | 0.175 | 0.064 |
| ADRA2A | Positive | -0.059 | 0.538 |
| DLC1 | Positive | -0.403 | <0.0001 |
| ITGA9 | Positive | -0.088 | 0.355 |
| SYTL4 | Positive | -0.183 | 0.053 |
| FRMD4B | Positive | -0.109 | 0.249 |
| HPSE2 | Positive | -0.093 | 0.328 |
| SETBP1 | Positive | 0.226 | 0.016 |
| PRKCH | Positive | 0.055 | 0.565 |
