# Supplementary material for: The m6A methyltransferase METTL14 promotes cell proliferation via SETBP1-mediated activation of PI3K-AKT signaling pathway in myelodysplastic neoplasms
Source: Leukemia. 2024 Jul 25;38(10):2246–58. doi: 10.1038/s41375-024-02350-3 (PMC11436359; doi:10.1038/s41375-024-02350-3)
Supplement: Supplementary file 4 — Supplementary Figure 1-4 [file 41375_2024_2350_MOESM4_ESM.docx]

**Supplementary Figures of the manuscript**

**Supplementary Figure 1**

**
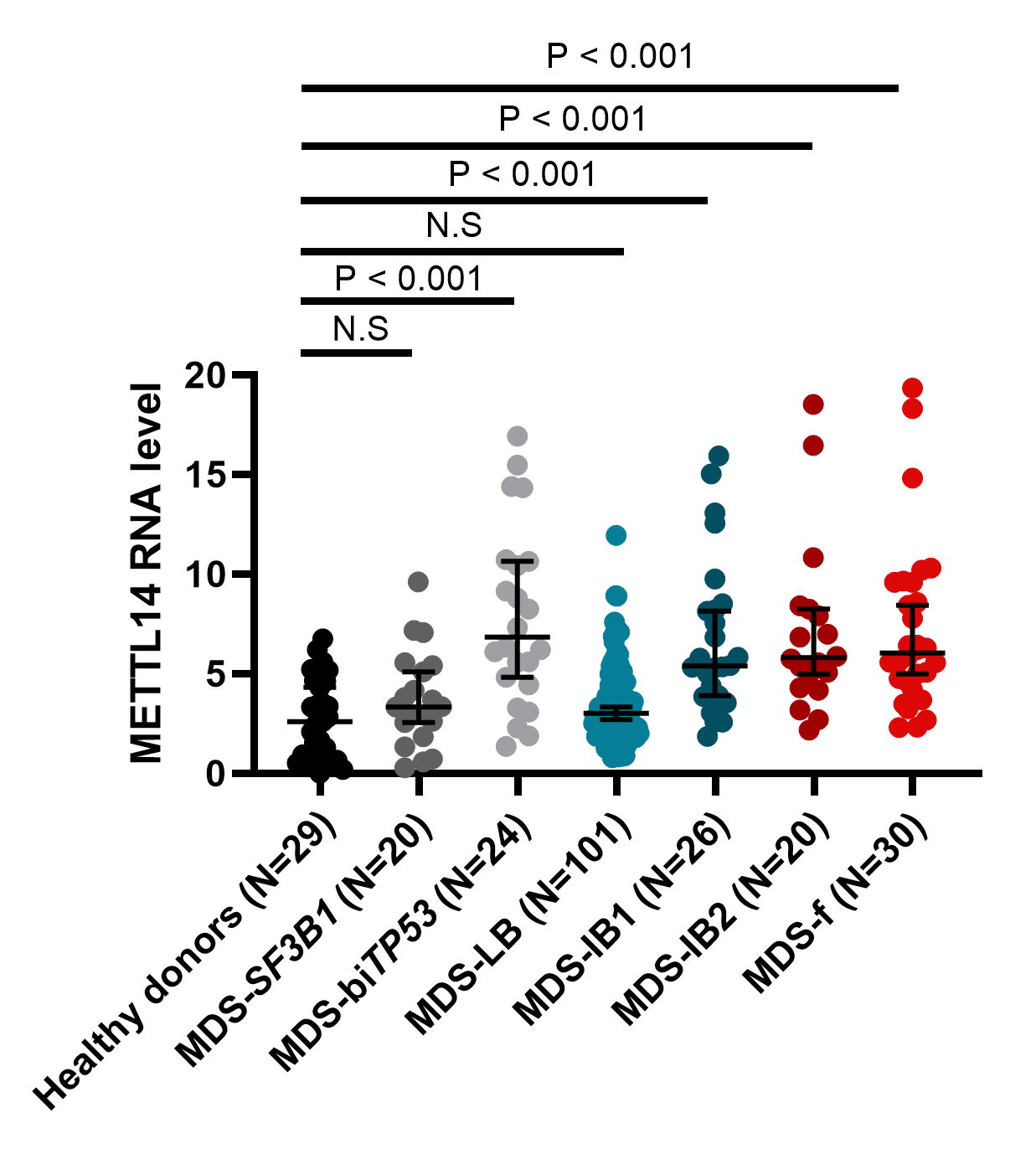
**

**Supplementary Figure 1. The expression of METTL14 in MDS subgroups according to 2022 WHO classification.**

**Supplementary Figure 2**


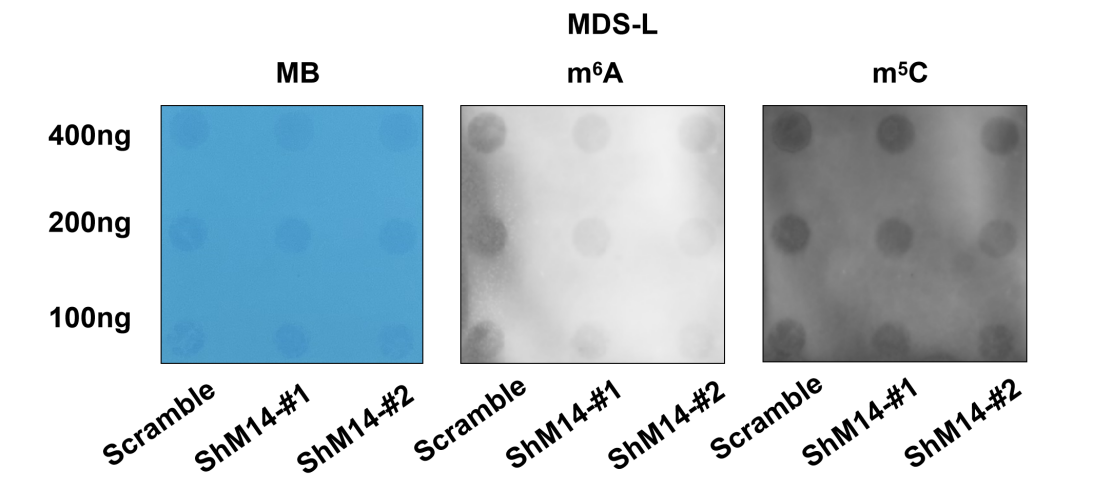


**Supplementary Figure 2.** **The level of RNA m^6^A and m^5^C modification in MDS-L cells with or without METTL14 knockdown.** Dot blot assays showing METTL14 knockdown in MDS-L cells led to obviously decreased m^6^A modification without significant alteration in m^5^C modification.

**Supplementary Figure 3**

**
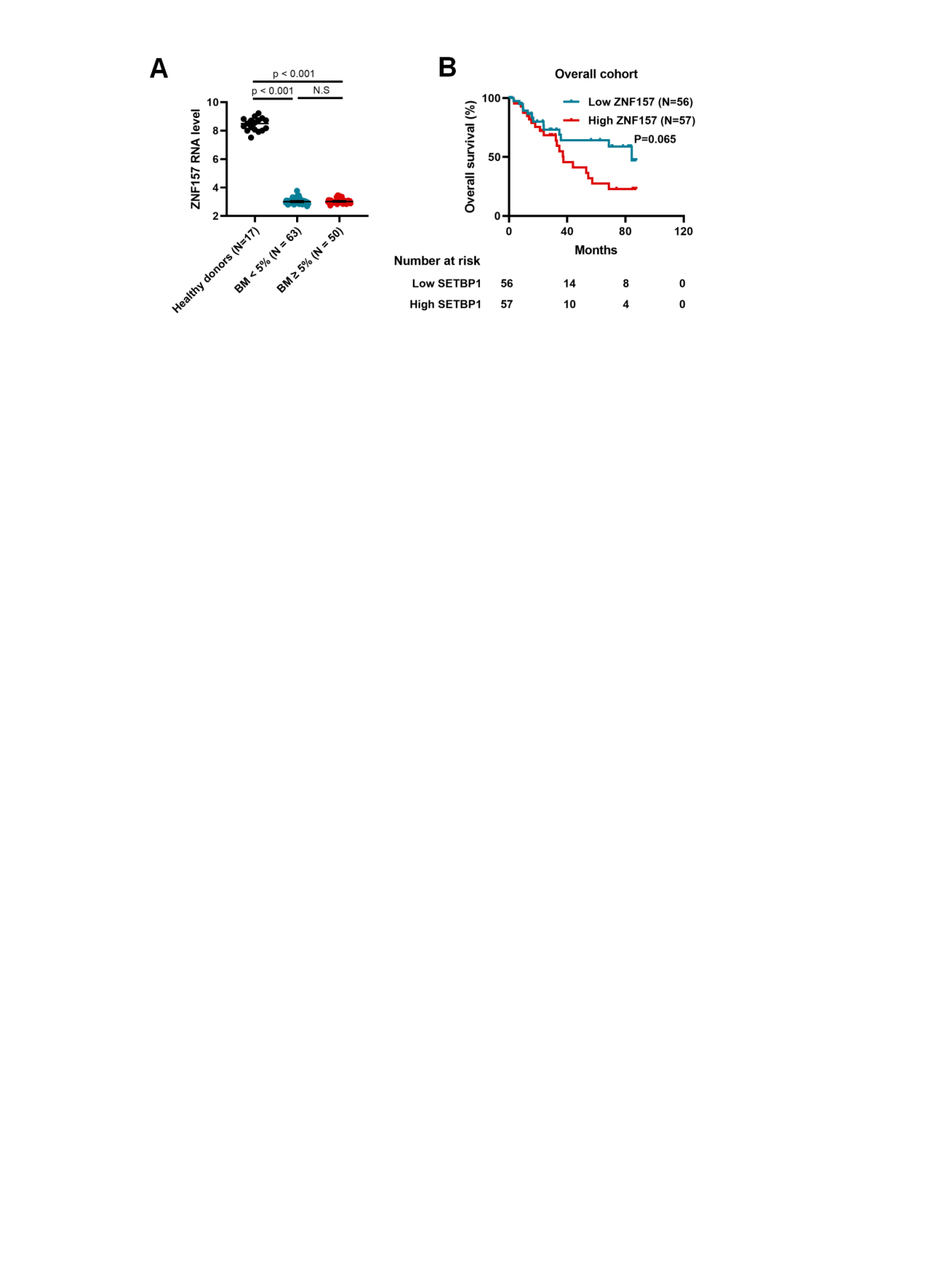
**

**Supplementary Figure 3. The clinical significance of ZNF157 in MDS based on the publicly available MDS database (GSE58831).**

1. ZNF157 expression in MDS subgroups based on the percentages of bone marrow blasts.
2. Kaplan–Meier survival analysis of ZNF157 level in MDS.

**Supplementary Figure 4.**


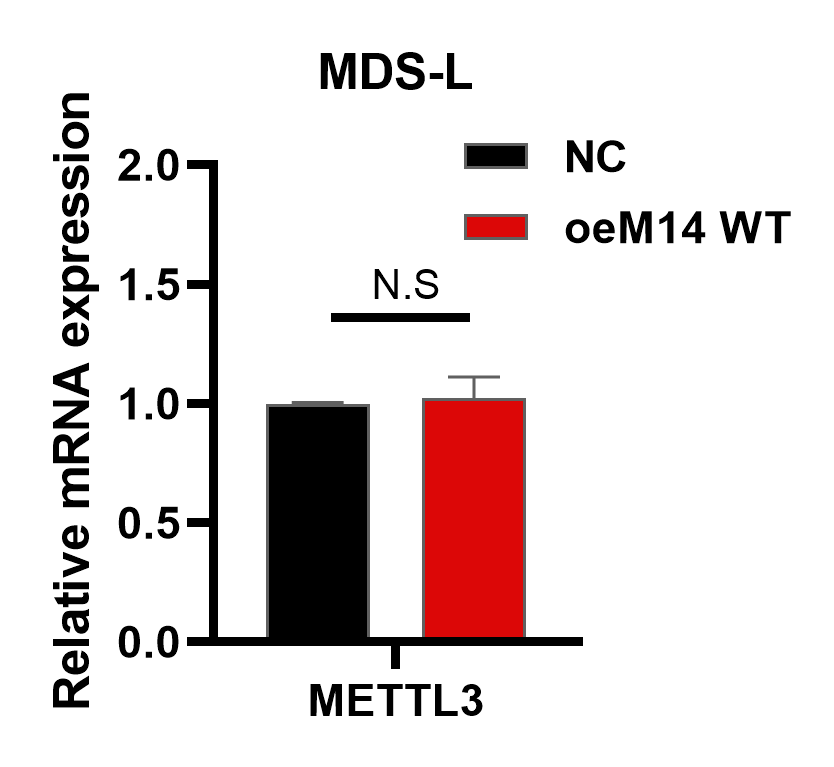


**Supplementary Figure 4. The expression of METTL3 mRNA in MDS-L cells with or without overexpression of METTL14 WT.** No significant alterations of METTL3 mRNA in MDS-L cells with overexpression of METTL14 WT indicated by q-PCR analysis.
